# Predicting or Pretending: Artificial Intelligence for Protein-Ligand Interactions Lack of Sufficiently Large and Unbiased Datasets

**DOI:** 10.3389/fphar.2020.00069

**Published:** 2020-02-25

**Authors:** Jincai Yang, Cheng Shen, Niu Huang

**Affiliations:** ^1^ School of Life Sciences, Peking University, Beijing, China; ^2^ National Institute of Biological Sciences, Beijing, China; ^3^ Graduate School of Peking Union Medical College, Chinese Academy of Medical Sciences, Beijing, China; ^4^ Tsinghua Institute of Multidisciplinary Biomedical Research, Tsinghua University, Beijing, China

**Keywords:** artificial intelligence, convolutional neural network, protein-ligand interaction, virtual screening, molecular docking, scoring function, topology fingerprint

## Abstract

Predicting protein-ligand interactions using artificial intelligence (AI) models has attracted great interest in recent years. However, data-driven AI models unequivocally suffer from a lack of sufficiently large and unbiased datasets. Here, we systematically investigated the data biases on the PDBbind and DUD-E datasets. We examined the model performance of atomic convolutional neural network (ACNN) on the PDBbind core set and achieved a Pearson R^2^ of 0.73 between experimental and predicted binding affinities. Strikingly, the ACNN models did not require learning the essential protein-ligand interactions in complex structures and achieved similar performance even on datasets containing only ligand structures or only protein structures, while data splitting based on similarity clustering (protein sequence or ligand scaffold) significantly reduced the model performance. We also identified the property and topology biases in the DUD-E dataset which led to the artificially increased enrichment performance of virtual screening. The property bias in DUD-E was reduced by enforcing the more stringent ligand property matching rules, while the topology bias still exists due to the use of molecular fingerprint similarity as a decoy selection criterion. Therefore, we believe that sufficiently large and unbiased datasets are desirable for training robust AI models to accurately predict protein-ligand interactions.

## Introduction

Structure-based virtual screening (molecular docking) has been widely used to discover new ligands based on target structures (Kitchen et al., 2004; Shoichet, 2004; Irwin and Shoichet, 2016; Zhou et al., 2016; Wang et al., 2017; Lyu et al., 2019; Peng et al., 2019). The molecular docking approach is designed to identify small molecules from a large chemical library that possess complementary to a protein binding site. The heart of molecular docking is the scoring function for estimation of binding affinities of protein-ligand complexes. Large research efforts in the field have been dedicated to the development of scoring functions in terms of their abilities to reproduce crystal ligand binding poses, to prioritize the known active compounds in a large compound database, and to predict the relative binding affinities (Stahl and Rarey, 2001; Halgren et al., 2004; Huang et al., 2006a; Wang et al., 2016; Liu et al., 2017; Guedes et al., 2018; Su et al., 2019). Despite some success, it is still very challenging to predict protein-ligand interactions accurately and efficiently using molecular docking.

In the retrospective studies, the performance of virtual screening was evaluated on several public available benchmarking datasets, including the Community Structure-Activity Resource (CSAR) (Dunbar et al., 2011), the PDBbind (Liu et al., 2017), the Directory of Useful Decoys (DUD) (Huang et al., 2006b), and the Directory of Useful Decoys - Enhanced (DUD-E) (Mysinger et al., 2012). The CSAR and PDBbind datasets were compiled to facilitate the prediction of the binding affinities based on experimental complex structures. The availability of experimental protein-ligand complex structures allows the structure-based featurization to correlate the protein-ligand binding interactions and the binding affinities. The DUD and DUD-E datasets were originally designed to assess docking enrichment performance by distinguishing the annotated actives from among a large database of computationally generated non-binding decoy molecules.

In recent years, deep learning (DL) technologies in the field of artificial intelligence (AI) have rapidly developed, and have been quickly introduced into the different aspects of drug discovery and development process (Chen et al., 2018; Ching et al., 2018; Hu et al., 2018; Ivanenkov et al., 2019; Xu et al., 2019; Zhavoronkov et al., 2019). However, DL relies on large and high-quality annotated datasets, and this approach is only in the early stages of applicability for protein-ligand binding prediction (Shen et al., 2019). Two types of representations have been applied in studying protein-ligand interactions (Ching et al., 2018). One is three-dimensional (3D) grid, which discretize protein-ligand complex structure into a 3D grid with features stored at the grid point (Wallach et al., 2015; Ragoza et al., 2017; Jiménez et al., 2018; Stepniewska-Dziubinska et al., 2018). For example, a 3D convolutional neural network (CNN) model was shown to outperform the AutoDock Vina in enrichment performance by achieving a mean area under the curve (AUC) of 0.86 on the DUD-E dataset (Ragoza et al., 2017). Another model (named Pafnucy) was tested for binding affinity prediction on the PDBbind v2013 core set with a Pearson R^2^ of 0.49 (Stepniewska-Dziubinska et al., 2018).

The other representation is graph neural network (Battaglia et al., 2018), every atom is a vertex and the atomic features (including atom type, charge, distances, and neighbors) in molecule are stored at the atom (Pereira et al., 2016; Gomes et al., 2017; Cang et al., 2018; Feinberg et al., 2018). For example, DeepVS was reported to achieve a mean AUC of 0.81 for cross-target cross validation (CV) on the DUD dataset (Pereira et al., 2016). The atomic convolutional neural network (ACNN) was developed for binding affinity prediction but did not outperform random forest (RF) on the PDBbind datasets (Gomes et al., 2017). Cang et al. (2018) achieved a Pearson R^2^ of 0.66 on the PDBbind v2013 core set using the model trained on the refined set.

However, Sieg et al. (2019) recently reported that the AI models were heavily biased by 1D properties and 2D topology trained on the DUD and DUD-E datasets. Only with the use of six physicochemical properties, RF classifiers achieved mean AUCs up to 1.0 for intra-target CV, while for cross-target CV on DUD and DUD-E, maximum mean AUCs of 0.78 and 0.80 were able to obtain, individually. Only using topology information of compounds, RF and DeepVS achieved a mean AUC of 0.78 for cross-target CV on DUD, and grid-based CNN model yielded a mean AUC of 0.84 for cross-target CV on DUD-E. Similarly, Chen et al. (2019) also reported the bias on topology in DUD-E. These studies demonstrate that AI models trained on ligand properties or ligand topology have comparable enrichment performance as those trained on docked complexes.

In the present work, we systematically investigated the data biases in the PDBbind and DUD-E datasets, including different data splitting methods, featurization, models, and metrics. We trained ACNN models (Gomes et al., 2017) on the protein-ligand complex structures, as well as on the ligand structures without the presence of proteins or on the protein structures by removing the ligand information. Strikingly, all these models performed comparably well in predicting binding affinities in test subsets, which strongly suggests that the ACNN models did not require learning essential protein-ligand interactions. Furthermore, we visualized the individual atomic contributions decomposed from the ACNN scores and found that the ACNN models may actually rely on the similarity of atomic features that exist in the training and test subsets to predict binding affinities. These results indicate that PDBbind has data biases in both proteins and ligands for building reliable AI models. Finally, we demonstrated that model learned the topology bias in DUD-E even after reducing the property bias by carefully designed CV experiments. We expect that our study will provide a useful guideline to assess the model performance in predicting protein-ligand interactions using state-of-the-art AI approaches.

## Methods

### Datasets

The PDBbind is a comprehensive collection of protein-ligand complexes in the Protein Data Bank (PDB) with experimentally measured binding affinities, which contains core, refined, and general sets (**Table 1**) (Li et al., 2014). For clarification, the PDBbind v2013 core set is identical to the v2015 core set. At present study, we only report the results obtained from the PDBbind v2015. The general set contains a total of 11,987 protein-ligand complexes in PDB with experimentally measured binding affinity data. The refined set contains 3,796 complex structures chosen from the general dataset to enforce higher quality protein-ligand complex structures and binding affinities. The core set consists of 195 high-quality complexes clustered in 65 structural groups, each containing three complexes with low, medium, and high binding affinities. In addition, Wan et al. (2013) modeled 2,431 binding interactions of 17 kinase inhibitors against 143 protein kinases using physics-based approach. We also tested the kinase inhibitor selectivity prediction on this dataset using ACNN models trained on the PDBbind refined set.

**Table 1:** The PDBbind and DUD-E datasets.

Name	Task type	Sets	Crystal structures	#Actives	#Decoys
**PDBbind**	Regression	Core	195	195	0
		Refined	3,706	3,706	0
		General	11,987	11,987	0
**DUD-E**	Classification	Original	102	22,886	1,411,214
		MW ≤ 500	102	19,374	1,182,039

The DUD and DUD-E datasets were designed for benchmarking molecular docking enrichment power by providing challenging decoys. For each annotated active, 50 decoys with six similar physicochemical properties, including molecular weight (MW) and cLogP, but dissimilar topology (fingerprint) were selected from the ZINC12 database (Irwin et al., 2012). The DUD-E dataset consists of 22,886 actives and 1,411,214 decoys against 102 targets. We compiled a variation of DUD-E, named DUD-E_(MW__≤__500)_ by simply removing actives with MW (only accounting for all heavy atoms) greater than 500 and the same fraction of decoys (**Table 1**).

### Dataset Splitting

Each PDBbind set was split into the training, validation, and test subsets following an 80/10/10 ratio. We trained models on the training subset by using early stopping to avoid overfitting, tuned hyperparameters on the validation subset to select the best model, and subsequently evaluated model performance on the test subset. We applied three types of dataset splitting methods, including random, ligand scaffold-based, and protein sequence-based splitting. Scaffold-based splitting was based on ligand scaffold similarity, where the ligand 2D scaffolds (Bemis and Murcko, 1996) were extracted using RDKit software (Landrum, 2006) and clustered using Extended-Connectivity Fingerprints (ECFP) (Rogers and Hahn, 2010) with Tanimoto coefficient (Tc) cutoff value of 0.8. The obtained large, medium, and small clusters were assigned into the training, validation, and test subsets, respectively. The test subset contained the smallest clusters to create a greater challenge for AI models. The sequence-based splitting was performed by using the UCLUST (Edgar, 2010) program with sequence identity cutoff of 0.4.

To stay consistent with a previous report, we trained models on the refined and general sets, and tested on the core set. To avoid the same protein-ligand complex used in training and testing simultaneously, we removed samples in the refined and general sets overlapping with the core set. In addition, we removed analogs or homologs based on ligand scaffold or protein sequence similarity when we applied scaffold-based and sequence-based splitting in training. Nevertheless, we subsampled the same number of samples (2,036 samples accounting for 55% of the refined set, 7,792 samples accounting for 65% of the general set) from the rest of samples in the refined or general sets, respectively, and split them into the training and validation subsets following a 90/10 ratio.

We split DUD-E into three folds based on target classes to perform the cross-class CV study. There are 26 kinases in the first fold, 31 targets in the second fold (including 15 proteases, 11 nuclear receptors, and five G-protein coupled receptors), and the rest of 45 targets in the third fold. We also applied a random CV on DUD-E by randomly splitting the targets into three folds with the same fold sizes as the cross-class CV.

### Models

#### ACNN

We applied the graph-based model ACNN implemented in the open source DeepChem package (Ramsundar et al., 2019) for predicting protein-ligand interactions in PDBbind. The ACNN model only requires atomic numbers and Cartesian coordinates of protein-ligand complexes as input to predict binding affinities. First, the ACNN model applies three independent atomic convolution blocks to extract atomic features from the ligand, protein, and protein-ligand complex, individually. In an atomic convolution block, the maximum number of closest neighbors (M) is used to represent the atomic environment for each atom. To represent the pairwise interaction, a radial basis function kernel is applied to map the distance between the atom and its each neighbor into a vector. And the atomic feature (a vector) is obtained by element-wise sum of M pairwise vectors. The atomic convolution blocks share the same initial parameters but will be changed after training. Secondly, one weight-sharing atomistic fully connected layer predicts atomic energies from all the atomic features. Thirdly, the ACNN model sums up the atomic energies to predict the energies of protein, ligand, and complex, individually, and then obtains the binding energy by subtracting the energies of protein and ligand from the energy of the binding complex. For analysis of bias in PDBbind, we modified ACNN to model only protein structures (protein alone), and only ligand structures (ligand alone) (**Supplementary Figure 1**). For protein alone, two independent atomic convolution blocks were used to extract atomic features from the same protein, and led to two different protein energies calculated from the same fully connected layer. The predicted “binding affinity” was the difference between two protein energies. The same strategy was applied for ligand alone as well. This strategy decouples the correlation of molecule size (number of atoms) and binding energy (sum of atomic energies), which enforces the ACNN model with the ability to learn atomic features.

All models were trained with an early-stopping strategy by stopping training if the performance on the validation subset did not improve in five epochs. The maximum number of neighbors of each atom was set to 4 at present study. We used a batch size of 16 and grouped samples with similar binding affinities into batches without changing the samples in one batch from the first to the last epochs. This training strategy is similar to the “curriculum learning” strategy (Bengio et al., 2009) because it reduces the difficulty of learning *via* training on the organized data.

#### Random Forest

Two feature sets for decoy selection were used to build the RF models (Breiman, 2001) to evaluate the bias in the DUD-E dataset. The first feature set consisted of six physicochemical properties, including MW (only accounting all heavy atoms), cLogP, number of rotatable bonds, number of hydrogen bond donors, number of hydrogen bond acceptors, and net charge. The second feature set was ECFP (Morgan fingerprint with a radius of 2 and 2,048 bits in RDKit), which has been widely applied to encode molecular 2D topology into fixed length binary vector. We computed the properties and ECFP using the open source RDKit package.

The RF classifier from scikit-learn (Pedregosa et al., 2011) version 0.21.3 was used. The default parameters were used except that the number of estimators was set to 100 and the seed of random state was set to 0 for deterministic behavior during fitting. The AUC value was used to evaluate the classification performance of the RF. The enrichment factor was calculated as EF_subset_ = (Actives_subset_/N_subset_)/(Actives_total_/N_total_). The higher the percentage of known actives found at a given percentage of the ranked database, the better the enrichment performance of the virtual screening. Since the practical value of virtual screening is to find active compounds as early as possible, we chose the enrichment factor at the top 1% of the ranked dataset (EF_1_) to evaluate the early enrichment performance in the present study. In kinase inhibitor selectivity prediction, we used predictive index (PI) as a semi-quantitative measurement of the power of the target ranking order, where PI value (ranging from 1 to −1) of 1 indicates the perfect prediction, and 0 is completely random (Pearlman and Charifson, 2001).

## Results

### High Performance Achieved on the PDBbind Datasets Using Random Splitting

**Figure 1:**
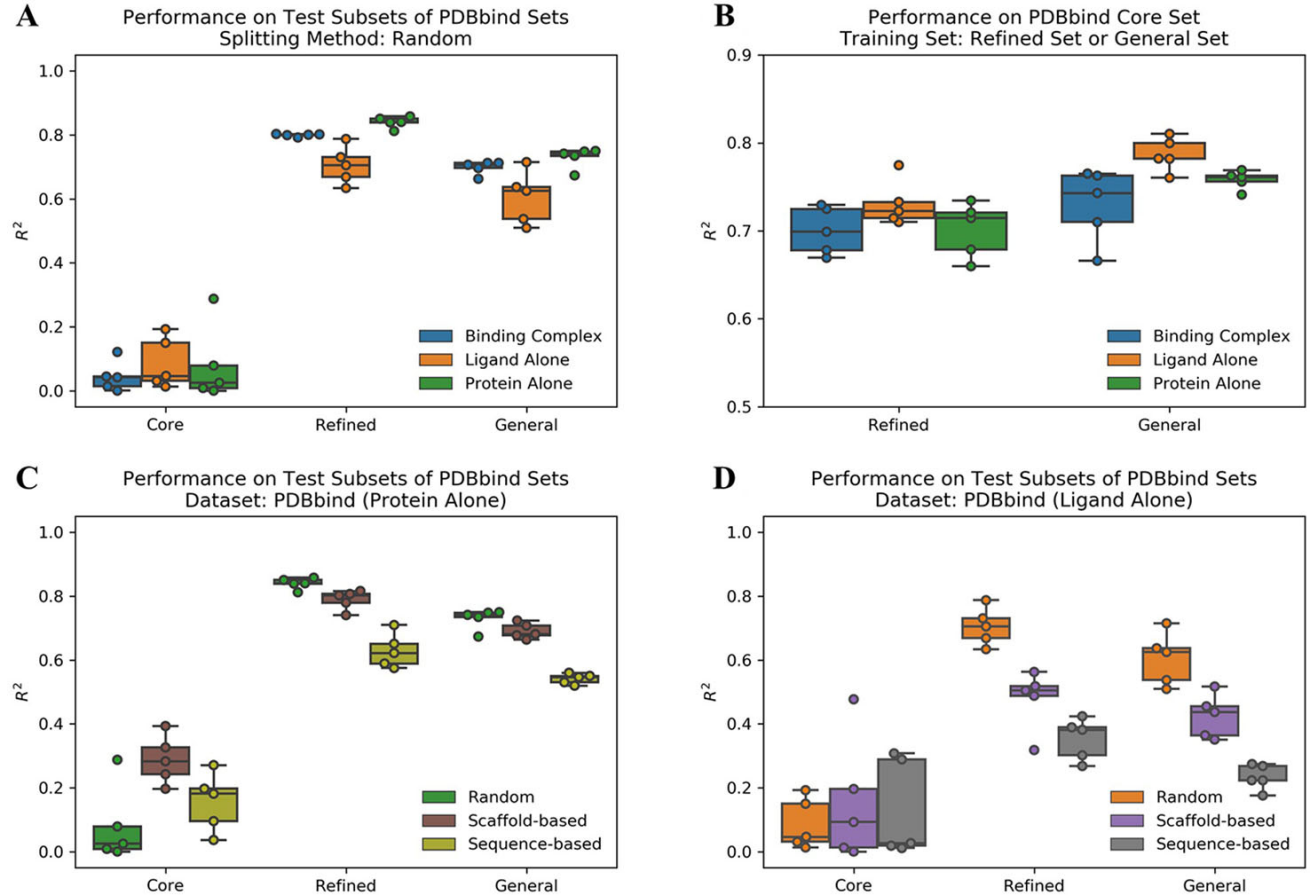
Atomic convolutional neural network performance measured by the Pearson R^2^ values obtained from the different PDBbind datasets using different splitting approaches. Each dataset was split into the training, validation, and test subsets five times with different random seeds following an 80/10/10 ratio, and studied on three different binding components, including protein-ligand complex structure (binding complex), only ligand structure (ligand alone), and only protein structure (protein alone), individually. **(A)** Models trained and tested within the same set. **(B)** Models trained on randomly selected subsets of the refined and the general sets (removing the core set structures) and tested on the core set. Models trained on the PDBbind datasets **(C)** (protein alone) and **(D)** (ligand alone) using different splitting methods.

We evaluated the performance of ACNN model to predict protein-ligand binding affinities on the PDBbind datasets using different data splitting approaches. The Pearson R^2^ values on test subsets are reported in **Supplementary Table 1**. Firstly, we used a random splitting approach to split each PDBbind dataset into the training, validation, and test subsets five times with different random seeds. The increased number of protein-ligand complexes in the refined and general sets improved the ACNN model performance significantly (**Figure 1A**). The core set had the lowest mean R^2^ value of 0.04, the refined and general sets with more samples were shown much higher performance with R^2^ values of 0.80 and 0.70, respectively. We also trained the models on the refined and general sets, and tested the models on the core set, individually. The results were also promising, outperformed previously reported results of R^2^ value of 0.66 using model trained on the refined set (Cang et al., 2018; Shen et al., 2019), with R^2^ values of 0.70 and 0.73 using models trained on the refined and general sets, individually (**Figure 1B** and **Supplementary Table 2**).

Since PDBbind contains large number of kinase targets (309 kinase structures accounting 9.76% of the refined set), we wanted to test the performance of ACNN model on a benchmarking dataset for kinase inhibitor selectivity modeling (Wan et al., 2013). Using the models trained on the PDBbind refined set, the calculated mean EF_20_ value of 1.12 and PI value of 0.01 indicate that such ACNN models cannot be used to predict the ranking order of the kinase targets for a given inhibitor (**Supplementary Table 3**).

To study the prediction power of the ACNN model, it is critical to decompose the contributions of the ligands and protein from the complex structure. Therefore, we generated two extra datasets by dividing the protein-ligand complex structure (binding complex) into ligand structure (ligand alone) and protein structure (protein alone), individually. Strikingly, the model performance did not change significantly on datasets of ligand alone or protein alone in both the refined and general sets (**Figure 1A, B** and **Supplementary Table 1**). These results indicate that the ACNN model does not require learning protein-ligand interactions to achieve high performance, and suggest that data biases exist in PDBbind, both with proteins and with ligands.

### Protein and Ligand Similarity Biases in PDBbind

Li et al. reported that the protein similarity impacts the performance of AI models (Li and Yang, 2017). Therefore, we applied sequence-based splitting to reduce the impact of the protein similarity between the training and test subsets. When trained on protein alone, the R^2^ value was reduced from 0.84 (random splitting) to 0.63 (sequence-based splitting) in the refined set; while it was reduced from 0.73 to 0.54 in the general set (**Figure 1C** and **Supplementary Table 1**). In addition, we guessed that ACNN learned the bias on ligand similarity. Therefore, we split the PDBbind datasets based on ligand scaffold similarity, and the performance of ACNN models was reduced significantly. When trained on ligand alone, the R^2^ value was reduced from 0.71 (random splitting) to 0.48 (scaffold-based splitting) in the refined set, and from 0.60 to 0.42 in the general set. Since similar targets bind similar ligands, it is not surprising that protein sequence-based splitting also significantly reduced the model performance compared to random splitting. The R^2^ values were reduced to 0.35 and 0.23 in the refined and general sets, individually (**Figure 1D** and **Supplementary Table 1**).

**Figure 2:**
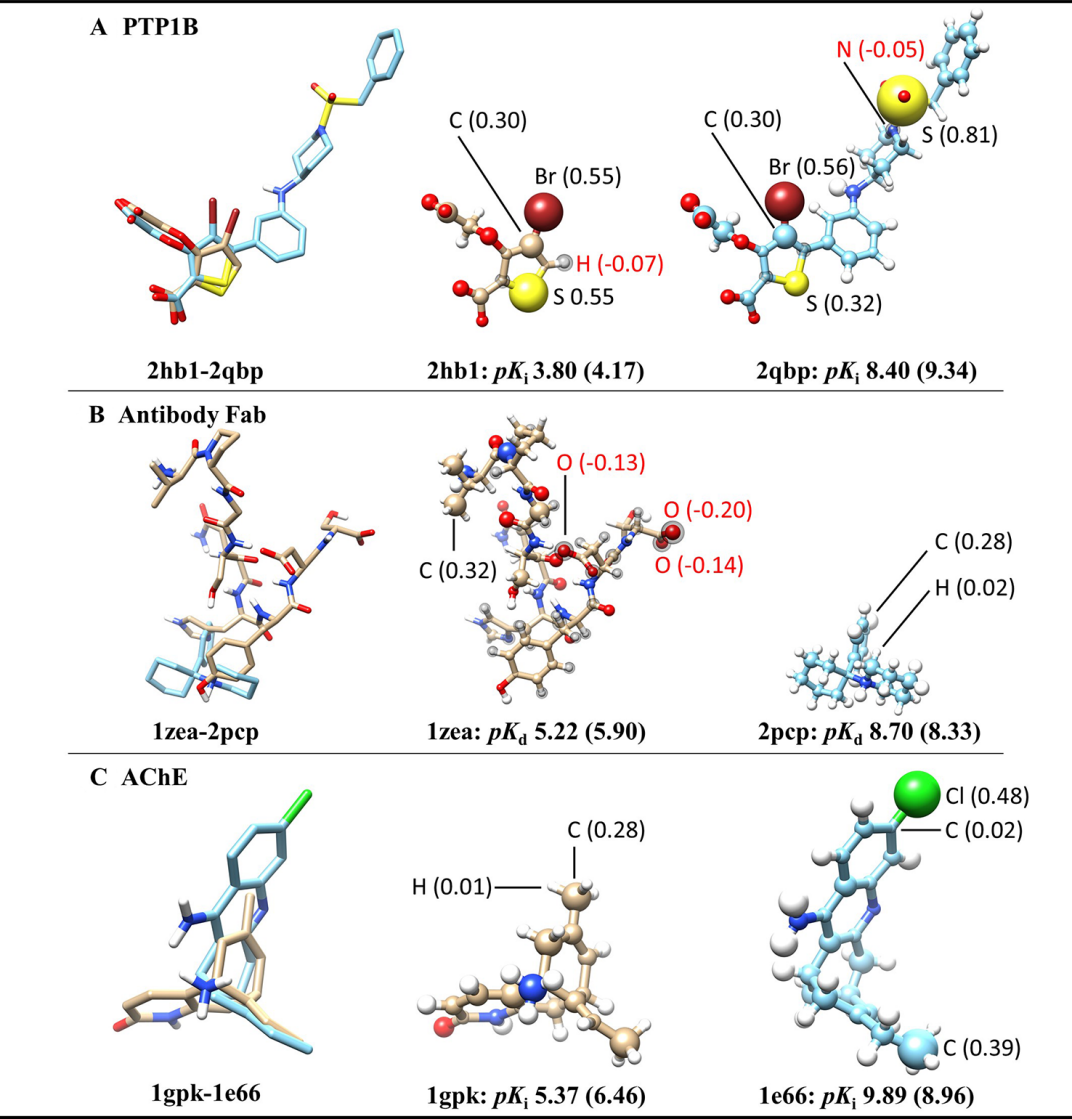
Atomic contributions derived from the ACNN model (ligand alone) on three representative systems chosen from the PDBbind core set, including **(A)** protein tyrosine phosphatase 1B (PTP1B) inhibitors, **(B)** ligands bound to the antibody Fab and **(C)** acetylcholinesterase (AChE) inhibitors. The ACNN model (ligand alone) was trained on the refined set (removing the core set structures) and tested on the core set. Each row shows two ligands from the same protein target with different binding affinities (*pK*_i_ or *pK_d_*) (predictive values included inside the parentheses). The first column shows the superimposed ligand structures using the binding pocket alignment approach. The second and third columns show atomic contributions of each ligand. The size of the balls represents the absolute values of atomic scores. The atomic scores of selected atoms are labeled explicitly. The atoms with black spheres have negative scores. The molecular images were generated using UCSF Chimera (Pettersen et al., 2004).

To further investigate what the ACNN model exactly learned from the ligand structures, we derived the atomic contributions from the ACNN models (ligand alone) trained on the PDBbind refined set (with structures in the core set removed) (**Figure 2**). Three representative systems were chosen from the core set to illustrate the atomic contributions of the ligands. Two protein tyrosine phosphatase 1B (PTP1B) inhibitors had similar atomic scores in Br atoms but different scores in S atoms, which suggests that the ACNN model could predict atomic contributions based on local atomic features (**Figure 2A**). However, the derived atomic contributions differed significantly in models trained with different random seeds, as demonstrated by the scores of the same Br atom changing from 0.55 to −0.04 in different models (**Supplementary Figure 2**). Atomic scores on the ligands bound to the antibody Fab showed that the model could predict one ligand (1zea) with larger molecular size but lower affinity by assigning negative scores on atoms with potentially unfavorable binding contributions (**Figure 2B**). For two acetylcholinesterase (AChE) inhibitors with similar size, the model correctly predicted the more potent inhibitor by identifying the presence of specific functional groups, such as Cl atom and ethyl group (**Figure 2C**). Combing the observations from those representative systems, the ACNN model is able to learn the correlation between atomic features and binding affinities. However, this correlation does not have to relate to protein-ligand interactions and may only represent the similarity of the ligands in PDBbind.

### Property Bias in DUD-E

**Figure 3:**
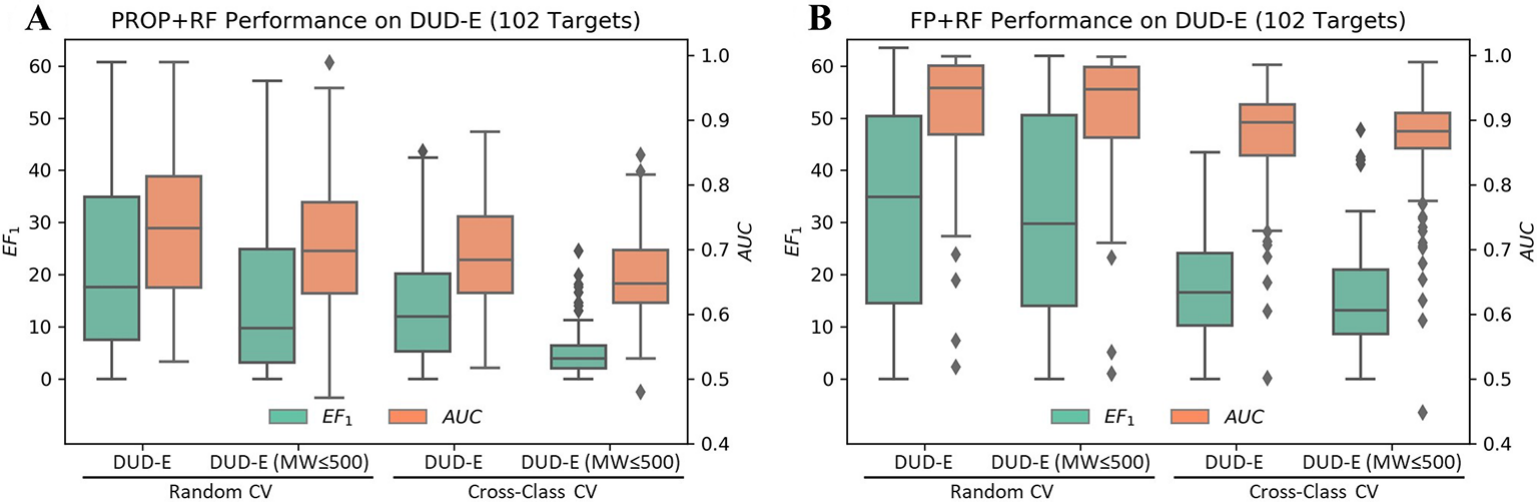
Performance of RF on the DUD-E datasets using **(A)** six properties or **(B)** topology fingerprints. Note that the DUD-E_(MW__≤__500)_ dataset was compiled by removing actives with MW (only including heavy atoms) greater than 500 and their associated decoys. The cross-class CV split the dataset into three folds based on target classes, and the random CV randomly split targets with the same fold sizes as in cross-class CV.

Although the accurate prediction of ligand binding affinities is the ultimate goal of molecular docking, the practical value of structure-based virtual screening is to enrich the active compounds in the top ranked subset. Generally, the success of a virtual screening method is evaluated by its capacity to discriminate known active compounds from a background of decoy molecules. However, Sieg et al. (2019) reported that the distributions of MW beyond 500 Da between actives and decoys in DUD-E were mismatched (**Supplementary Figure 3**). Indeed, only using six properties as features, RF achieved a mean EF_1_ of 22.2 and a mean AUC of 0.73 in random CV on DUD-E (**Figure 3A**). Therefore, we compiled the DUD-E_(MW__≤__500)_ dataset to remove this specific MW bias (**Supplementary Figure 4**). A mean EF_1_ of 15.4 and a mean AUC of 0.71 was achieved in random CV on DUD-E_(MW__≤__500)_, more importantly, a mean EF_1_ of 5.14 and a mean AUC of 0.66 was achieved in cross-class CV, which indicates that the model cannot use property bias to achieve high performance in cross-class CV on the DUD-E_(MW__≤__500)_.

### Topology Bias in DUD-E

In DUD and DUD-E, the actives and decoys against the same target are dissimilar on topology and can be easily differentiated based on fingerprint (von Korff et al., 2009; Venkatraman et al., 2010; Hu et al., 2012; Lagarde et al., 2015; Kearnes et al., 2016; Sieg et al., 2019). However, whether the actives and decoys can be differentiated in cross-target CV based on fingerprint remains unclear, due to the mixed property bias and topology bias. By avoiding the use of property bias, we may study the independent contribution of topology bias on DUD-E. As shown in **Figure 3B**, using RF with molecular fingerprint (FP) as features, a mean AUC of 0.91 and a mean EF_1_ of 32.75 in random CV was obtained on DUD-E. The model achieved a mean AUC of 0.86 and a mean EF_1_ of 15.33 in cross-class CV on the DUD-E_(MW__≤__500)_. These results indicate that the model can still use topology bias in DUD-E even after avoiding the property bias.

**Figure 4:**
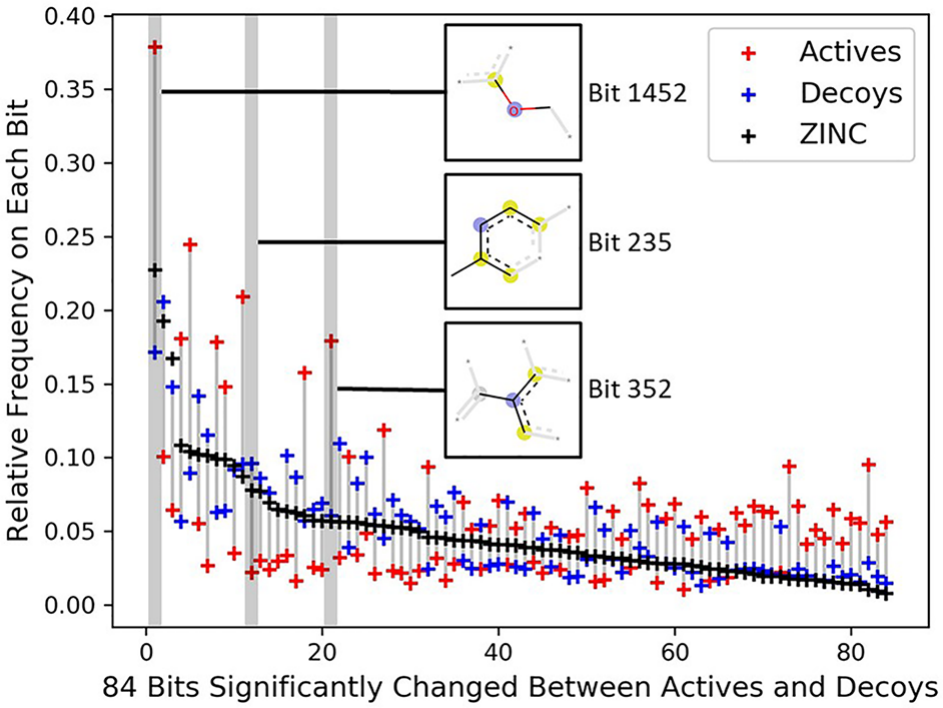
Significantly changed bits between actives and decoys on DUD-E_(MW__≤__500)_. Eighty-four bits with absolute log2 fold change ≥ 1 between the actives and decoys and mean relative frequency ≥ 0.03 were selected as representative bits from the Morgan fingerprints (2,048 bits). The bits were sorted by frequencies of ZINC12 compounds. The chemical features of three selected bits are presented, and the chemical features of all 84 bits are summarized in **Supplementary Table 4**.

To investigate the topology bias in the DUD-E dataset, we calculated the relative frequency of bit set on each bit (2,048 bits) for actives and decoys in DUD-E_(MW__≤__500)_ and the bit frequencies of ZINC12 compounds as reference (**Supplementary Figure 5**). Eighty-four bits with absolute log2 fold change ≥ 1 and mean relative frequency ≥ 0.03 were selected as representative bits (**Supplementary Figure 6**). About half of bit frequencies of actives and decoys are located on the opposite side of the bit frequencies of ZINC12 compounds, for example, the most populated bit 1,452 representing an aryl-alkyl ether group (**Figure 4**). This indicates that the topology distribution of decoys is strikingly different to actives. The rest of representative bits have relatively close frequencies between decoys and ZINC12 compounds, while larger differences between actives and ZINC12 compounds exist, such as bit 235 (representing six-membered aromatic ring) and bit 352 (representing aromatic ring with a sp2-hybridized carbon substituent). This further demonstrates that topology bias is not only caused by using fingerprint as a decoy filter, but also resulted from the different topology distribution between actives and ZINC12 compounds. Therefore, the DUD and DUD-E datasets are not suitable for training models which directly or indirectly utilize the compound topological information.

## Discussion and Conclusions

State-of-the-art AI technologies represent a new paradigm in virtual screening with both opportunities and challenges for future improvement. The differences in different AI models mainly come from two aspects: one is the training dataset, and the other is the characterization method. At present work, we focused on analyzing the biases in two widely applied datasets for protein-ligand interactions. The former is represented by PDBbind, a collection of experimentally determined protein-ligand complex structures with known binding affinities, which is reliable, but the amount of data is small and arguably suffers from the data redundancy caused by the protein and ligand similarity. Our systematic investigation of ACNN models on the PDBbind datasets led to a surprising observation that the model performance was not correlated with learning essential protein-ligand interactions. Even the models trained on ligands or proteins performed as well as trained on complexes, while data splitting based on the similarity (protein sequence or ligand scaffold) clustering reduced the performance significantly. This suggests that the model performance may rely on the similarity of atomic features existing in the training and test subsets. It is expected that the rapidly increased amount of protein-ligand binding and structural data will improve the generality of the models by sampling the much larger and diverse chemical space.

DUD-E has become a common dataset for evaluating structure-based virtual screening methods, which were designed to benchmark enrichment performance by prioritizing the actives among a large amount of property-match but topology-dissimilar decoy molecules. As evidenced at present study, the topology bias is difficult to avoid when train on DUD-E. Therefore, care must be taken when using DUD-E for training AI models to predict protein-ligand interactions. However, DUD-E can still serve as an independent dataset to test the prediction power of AI models without using it for training. The use of fingerprint for selecting topological dissimilar decoys in the DUD and DUD-E datasets introduces topology bias in cross-target, and even cross-class CV. If we want to perform cross-target CV on DUD-like datasets for benchmarking AI models, the decoys shall be selected not only dissimilar to actives of a specific target, but also similar to actives of the other targets. Therefore, it is desirable to develop a more sophisticated approach for DUD-like decoy selection by depleting the topology bias, and such dataset may serve as a general-purpose benchmarking dataset to assess the enrichment performance of different virtual screening approaches (including AI models).

Nevertheless, it is encouraging that ACNN models have shown powerful capability for learning correlations hidden in structural data. Using the same neural network structure, ACNN was able to learn the structural similarities between ligands and between proteins. Even after protein sequence similarity clustering, ACNN still performed well in predicting ligand binding affinities. It is likely that ACNN model is well suitable for analysis of protein binding pocket, and it can be applied in protein pocket similarity analysis and protein pocket druggability prediction.

In summary, sufficiently large and unbiased datasets are desirable to fully exploit the potential of AI models for protein-ligand interactions. In addition to the guidelines proposed by Sieg et al. (2019), we can envision extra practical guidelines in developing and applying AI-based models. First of all, target structure-based methods do not guarantee that the performance of predicting ligand binding affinities is correlated with the learning of protein-ligand interactions. Vice versa, we demonstrated that ACNN models trained on the PDBbind datasets did not learn the essential protein-ligand interactions. Therefore, control experiments of training on the free ligands (ligand alone) and the free proteins (protein alone) can facilitate our understanding of what the AI models learned from the complex structures. Secondly, PDBbind is probably still going to be the best quality and the most accessible dataset for benchmarking protein-ligand interactions. However, it is necessary to evaluate the model performance by splitting datasets based on protein sequence and ligand scaffold similarity. Redundancy reduction increases the level of difficulty in model training, but will definitely improve the robustness of model transferability. Lastly, protein-ligand binding follows the laws of physics. The interpretability of AI models is critical for studying protein-ligand binding interactions, and visualization of atomic contributions decomposed from the models shall be engaged in extracting human understandable insights.

## Data Availability Statement

All scripts and user tutorial are available at https://github.com/hnlab/can-ai-do. The kinase inhibitors dataset is available at http://www.huanglab.org.cn/kinome/kinome-ligand.tgz.

## Author Contributions

NH and JY designed the project. JY and CS performed the computational studies. JY and NH analyzed the data and wrote the manuscript. All authors discussed the results and commented on the manuscript.

## Funding

Computational support was provided by the Special Program for Applied Research on Super Computation of the NSFC-Guangdong Joint Fund (the second phase) under Grant No. U1501501 to NH.

## Conflict of Interest

The authors declare that the research was conducted in the absence of any commercial or financial relationships that could be construed as a potential conflict of interest.
