# Supplementary material for: Predicting or Pretending: Artificial Intelligence for Protein-Ligand Interactions Lack of Sufficiently Large and Unbiased Datasets
Source: Front Pharmacol. 2020 Feb 25;11:69. doi: 10.3389/fphar.2020.00069 (PMC7052818; doi:10.3389/fphar.2020.00069)
Supplement: Supplementary file 1 [file DataSheet_1.pdf]

Supplementary Table 1. Performance of ACNN models on the test subsets of the PDBbind (five random seeds). The averaged Pearson R^2 values and standard errors are reported.

Splitting method	Binding component	Core	Refined	General
Random	Complex	0.04 (±0.05)	0.80 (±0.00)	0.70 (±0.02)
	Ligand alone	**0.09** (±0.08)	0.71 (±0.06)	0.60 (±0.08)
	Protein alone	0.08 (±0.12)	**0.84** (±0.02)	**0.73** (±0.03)
Scaffold	Complex	0.14 (±0.12)	0.60 (±0.04)	0.61 (±0.03)
	Ligand alone	0.16 (±0.20)	0.48 (±0.09)	0.42 (±0.07)
	Protein alone	**0.29** (±0.08)	**0.79** (±0.03)	**0.69** (±0.02)
Sequence	complex	0.08 (±0.13)	0.54 (±0.06)	0.47 (±0.04)
	Ligand alone	0.13 (±0.15)	0.35 (±0.07)	0.23 (±0.04)
	Protein alone	**0.16** (±0.09)	**0.63** (±0.05)	**0.54** (±0.02)

Supplementary Table 2. Performance of ACNN model on PDBbind core set (five random seeds). The averaged Pearson R^2 values and standard errors are reported.

**Splitting method**	**Binding component**	**Training set (refined)**	**Training set (general)**
Random	Complex	0.70 (±0.03)	0.73 (±0.04)
	Ligand alone	**0.73** (±0.03)	**0.79** (±0.02)
	Protein alone	0.70 (±0.03)	0.76 (±0.01)
Scaffold	Complex	0.69 (±0.02)	**0.76** (±0.03)
	Ligand alone	**0.77** (±0.02)	0.76 (±0.01)
	Protein alone	0.67 (±0.02)	0.69 (±0.04)
Sequence	Complex	0.64 (±0.05)	0.71 (±0.03)
	Ligand alone	**0.75** (±0.03)	**0.73** (±0.02)
	Protein alone	0.63 (±0.05)	0.73 (±0.02)

Supplementary Table 3. Performance of ACNN model on a kinase inhibitor dataset. The averaged EF_{20} , PI values and standard errors are reported.

Inhibitor (HET ID)	Physics-based approach [a]		ACNN [b]	
	EF_{20} [c]	PI [d]	EF_{20}	PI
VX-745 (52P)	**5.00**	0.60	0.00 (±0.00)	-0.10 (±0.18)
Gefitinib (IRE)	5.00	0.57	0.51 (±1.13)	-0.07 (±0.11)
BI-2536 (R78)	5.00	0.38	2.03 (±1.13)	0.13 (±0.14)
LY-333531 (LY4)	5.00	0.35	2.01 (±2.76)	0.08 (±0.13)
CP-690550 (MI1)	3.80	0.59	1.28 (±0.90)	0.19 (±0.19)
Erlotinib (AQ4)	3.04	0.44	1.42 (±0.91)	-0.08 (±0.08)
Dasatinib (1N1)	2.75	**0.64**	0.97 (±0.44)	0.10 (±0.09)
Vandetanib (ZD6)	2.32	0.25	0.84 (±0.39)	0.03 (±0.07)
A-674563 (SS3)	2.27	0.45	**2.61** (±0.51)	**0.37** (±0.04)
Flavopiridol (CPB)	1.70	0.07	1.70 (±1.20)	0.15 (±0.11)
VX-680 (VX6)	1.69	0.29	1.01 (±0.47)	-0.01 (±0.07)
Sunitinib (B49)	1.68	0.04	1.31 (±0.37)	0.07 (±0.09)
TAE-684 (GUI)	1.61	0.13	0.84 (±0.06)	-0.08 (±0.09)
Staurosporine (STU)	1.32	0.29	1.22 (±0.14)	0.03 (±0.10)
Crizotinib (VGH)	1.28	0.31	0.00 (±0.00)	-0.19 (±0.09)
SB-203580 (SB2)	1.25	0.15	0.75 (±1.12)	-0.21 (±0.18)
R406 (585)	1.21	0.30	0.61 (±0.15)	-0.22 (±0.06)
Mean	2.70	0.34	1.12 (±1.09)	0.01 (±0.18)

[a] Results from (Wan et al., 2013). [b] ACNN models (binding complex) trained on PDBbind refined set (with removal of structures overlapping with core set). [c] Number of predicted targets in top 20% denotes the number of true positives in top 20% of ranked kinase targets, which corresponds to the enrichment factors (EF_{20}) compared to random selection. [d] PI is semi-quantitative measurement of the correlation of predicted binding affinity with experimental values.

Supplementary Table 4. Visualization of fingerprint bits.

	bit	zinc_freq	log2_FC	mean_freq	active_freq	decoy_freq	example_1	example_2	example_3	example_4
1	bit: 1452	zinc_freq: 22.7%	log2(FC): 1.14	mean_freq: 27.5%	active_freq: 37.9%	decoy_freq: 17.2%	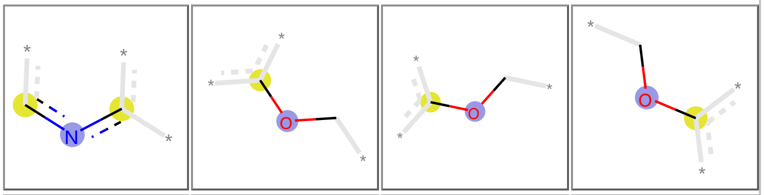			
2	bit: 283	zinc_freq: 19.2%	log2(FC): -1.03	mean_freq: 15.3%	active_freq: 10.0%	decoy_freq: 20.6%	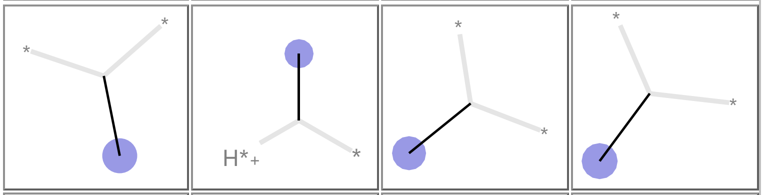			
3	bit: 41	zinc_freq: 16.7%	log2(FC): -1.20	mean_freq: 10.6%	active_freq: 6.4%	decoy_freq: 14.8%	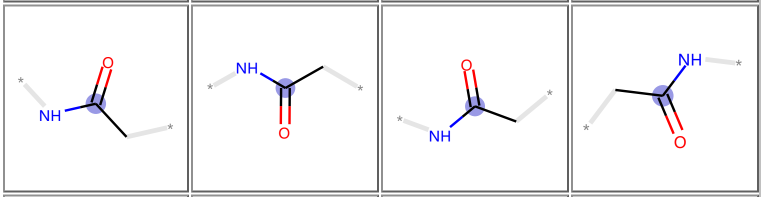			
4	bit: 1866	zinc_freq: 10.8%	log2(FC): 1.67	mean_freq: 11.9%	active_freq: 18.1%	decoy_freq: 5.7%	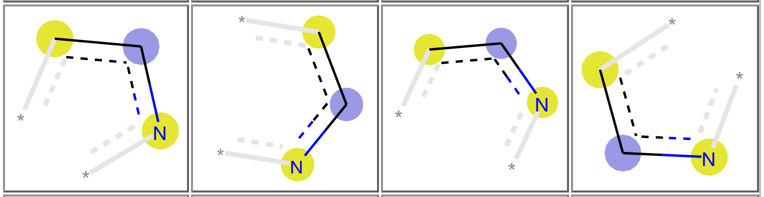			
5	bit: 1160	zinc_freq: 10.4%	log2(FC): 1.45	mean_freq: 16.7%	active_freq: 24.5%	decoy_freq: 8.9%	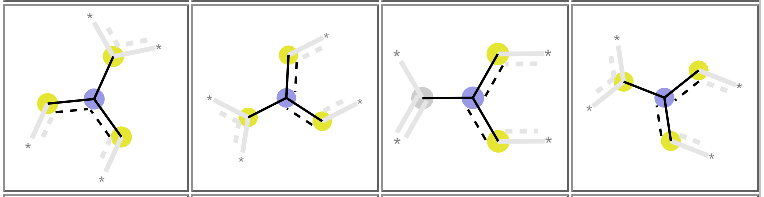			
6	bit: 116	zinc_freq: 10.2%	log2(FC): -1.36	mean_freq: 9.8%	active_freq: 5.5%	decoy_freq: 14.2%	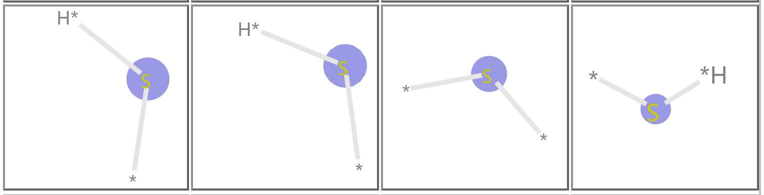			
7	bit: 1823	zinc_freq: 10.1%	log2(FC): -2.12	mean_freq: 7.1%	active_freq: 2.6%	decoy_freq: 11.5%	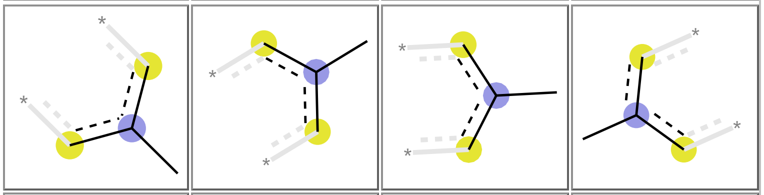			
8	bit: 1535	zinc_freq: 9.9%	log2(FC): 1.51	mean_freq: 12.0%	active_freq: 17.8%	decoy_freq: 6.3%	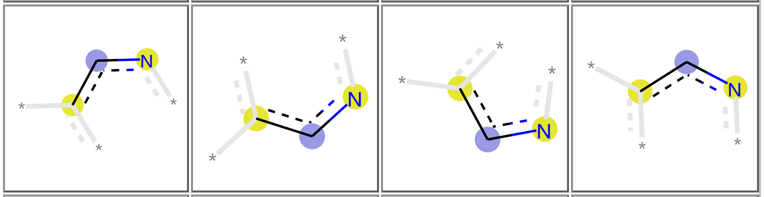			
9	bit: 1066	zinc_freq: 9.9%	log2(FC): 1.20	mean_freq: 10.6%	active_freq: 14.8%	decoy_freq: 6.4%	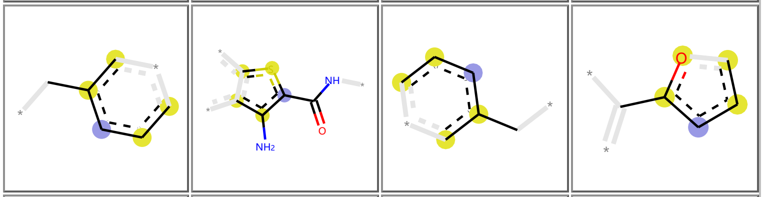			
10	bit: 1238	zinc_freq: 9.4%	log2(FC): -1.38	mean_freq: 6.3%	active_freq: 3.5%	decoy_freq: 9.2%	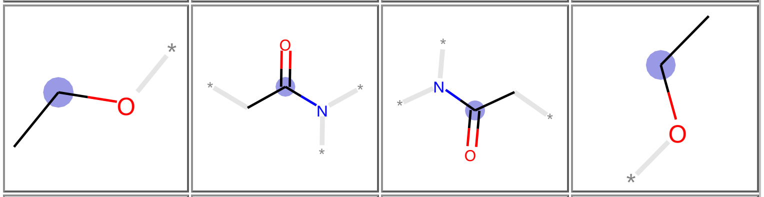			
11	bit: 1357	zinc_freq: 8.7%	log2(FC): 1.13	mean_freq: 15.2%	active_freq: 20.9%	decoy_freq: 9.6%	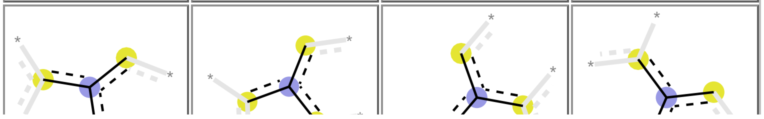			

	bit	zinc_freq	log2_FC	mean_freq	active_freq	decoy_freq	
							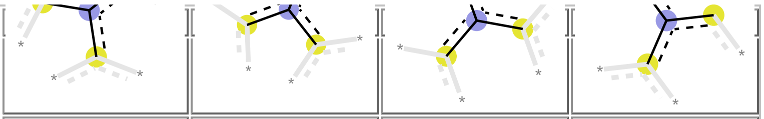
12	bit: 235	zinc_freq: 7.8%	log2(FC): -2.13	mean_freq: 5.9%	active_freq: 2.2%	decoy_freq: 9.6%	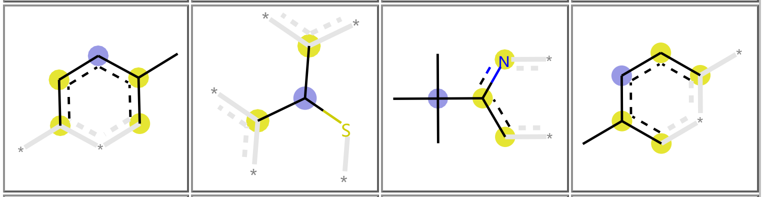
13	bit: 1164	zinc_freq: 7.7%	log2(FC): -1.52	mean_freq: 5.8%	active_freq: 3.0%	decoy_freq: 8.6%	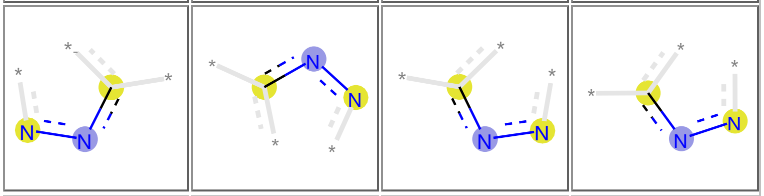
14	bit: 145	zinc_freq: 6.9%	log2(FC): -1.67	mean_freq: 5.0%	active_freq: 2.4%	decoy_freq: 7.6%	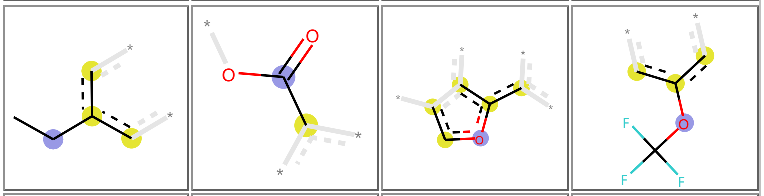
15	bit: 147	zinc_freq: 6.5%	log2(FC): -1.13	mean_freq: 4.7%	active_freq: 3.0%	decoy_freq: 6.5%	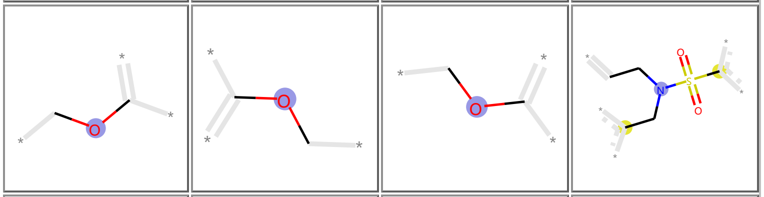
16	bit: 1309	zinc_freq: 6.4%	log2(FC): -1.59	mean_freq: 6.7%	active_freq: 3.4%	decoy_freq: 10.1%	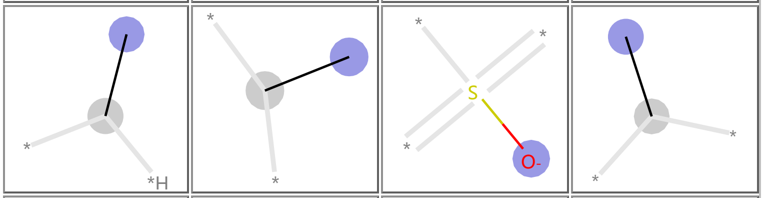
17	bit: 341	zinc_freq: 6.2%	log2(FC): -2.43	mean_freq: 5.1%	active_freq: 1.6%	decoy_freq: 8.7%	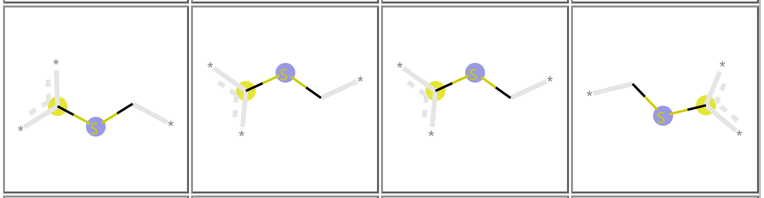
18	bit: 896	zinc_freq: 6.1%	log2(FC): 1.47	mean_freq: 10.7%	active_freq: 15.8%	decoy_freq: 5.7%	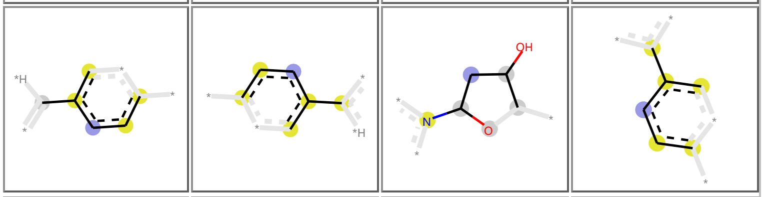
19	bit: 753	zinc_freq: 5.7%	log2(FC): -1.34	mean_freq: 4.5%	active_freq: 2.6%	decoy_freq: 6.5%	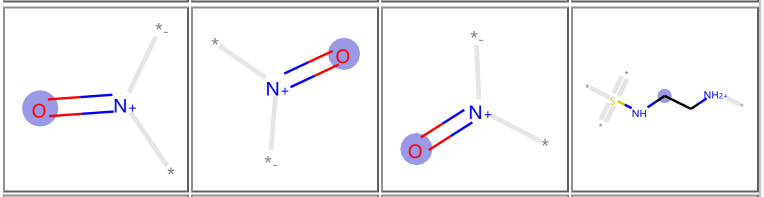
20	bit: 747	zinc_freq: 5.7%	log2(FC): -1.53	mean_freq: 4.6%	active_freq: 2.4%	decoy_freq: 6.9%	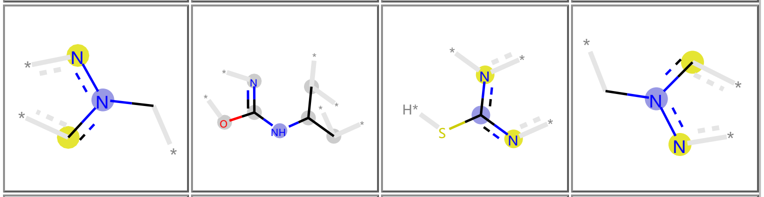
21	bit: 352	zinc_freq: 5.7%	log2(FC): 1.57	mean_freq: 12.0%	active_freq: 17.9%	decoy_freq: 6.0%	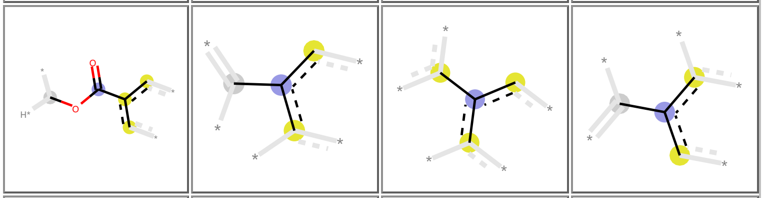
22	bit: 728	zinc_freq: 5.6%	log2(FC): -1.77	mean_freq: 7.1%	active_freq: 3.2%	decoy_freq: 11.0%	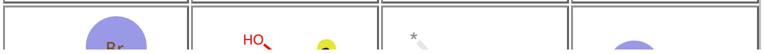

	bit	zinc_freq	log2_FC	mean_freq	active_freq	decoy_freq	
							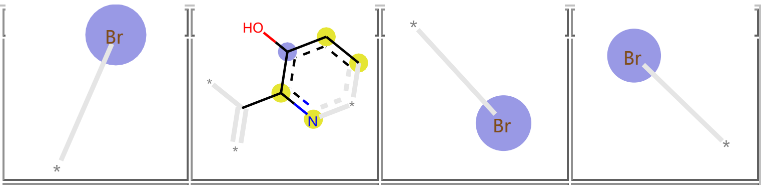
23	bit: 102	zinc_freq: 5.6%	log2(FC): 1.37	mean_freq: 7.0%	active_freq: 10.1%	decoy_freq: 3.9%	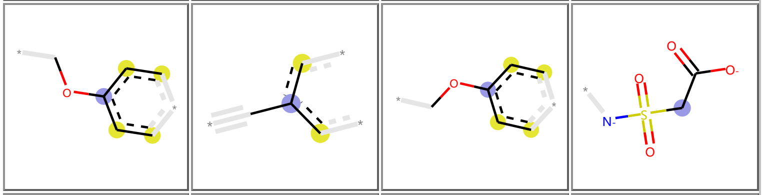
24	bit: 725	zinc_freq: 5.6%	log2(FC): -1.29	mean_freq: 5.8%	active_freq: 3.4%	decoy_freq: 8.2%	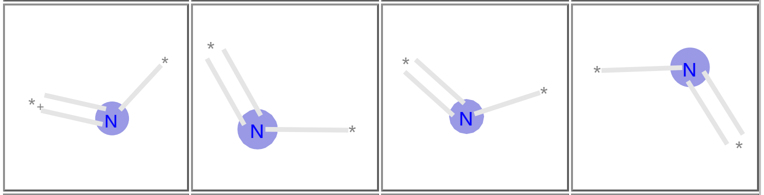
25	bit: 1279	zinc_freq: 5.5%	log2(FC): -1.04	mean_freq: 7.4%	active_freq: 4.9%	decoy_freq: 10.0%	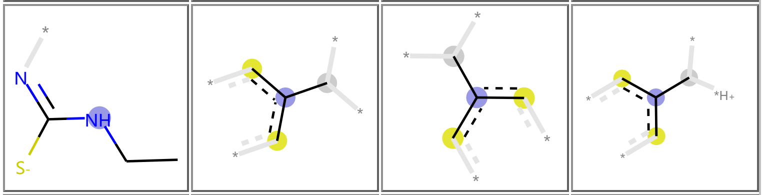
26	bit: 1195	zinc_freq: 5.4%	log2(FC): -1.55	mean_freq: 4.1%	active_freq: 2.1%	decoy_freq: 6.2%	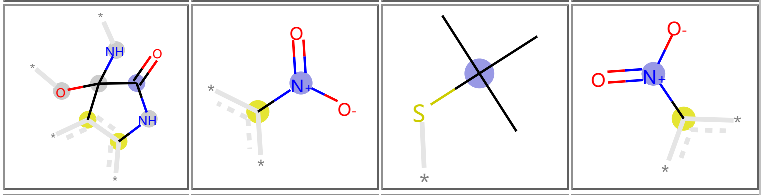
27	bit: 736	zinc_freq: 5.3%	log2(FC): 1.39	mean_freq: 8.2%	active_freq: 11.9%	decoy_freq: 4.5%	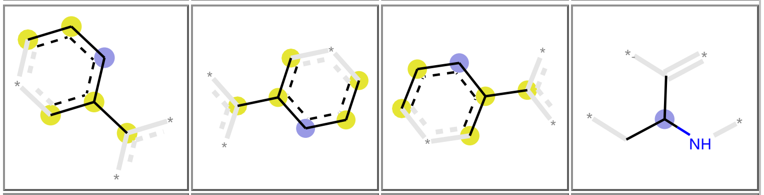
28	bit: 237	zinc_freq: 5.3%	log2(FC): -1.62	mean_freq: 4.7%	active_freq: 2.3%	decoy_freq: 7.1%	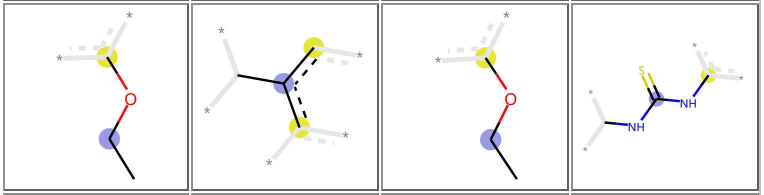
29	bit: 1963	zinc_freq: 5.2%	log2(FC): -1.50	mean_freq: 4.1%	active_freq: 2.1%	decoy_freq: 6.1%	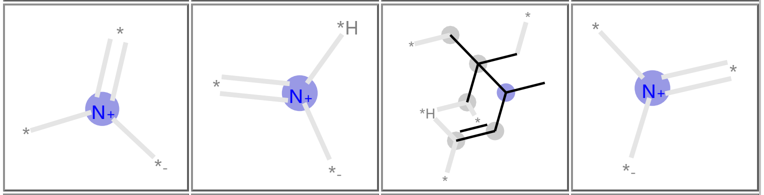
30	bit: 1013	zinc_freq: 5.2%	log2(FC): -1.95	mean_freq: 3.6%	active_freq: 1.5%	decoy_freq: 5.7%	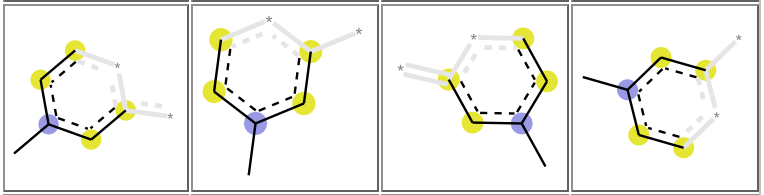
31	bit: 1920	zinc_freq: 5.0%	log2(FC): -1.15	mean_freq: 3.7%	active_freq: 2.3%	decoy_freq: 5.2%	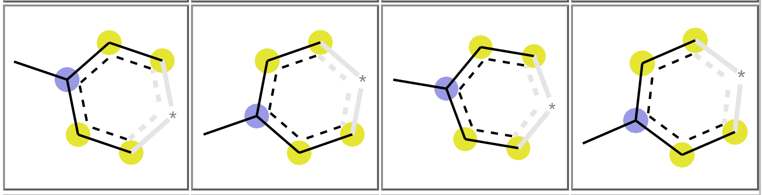
32	bit: 1603	zinc_freq: 4.6%	log2(FC): 1.94	mean_freq: 5.9%	active_freq: 9.4%	decoy_freq: 2.4%	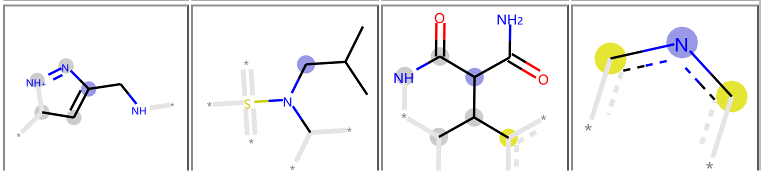

	bit	zinc_freq	log2_FC	mean_freq	active_freq	decoy_freq	
							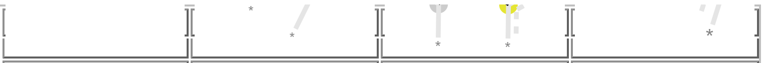
33	bit: 257	zinc_freq: 4.6%	log2(FC): -1.09	mean_freq: 4.9%	active_freq: 3.1%	decoy_freq: 6.7%	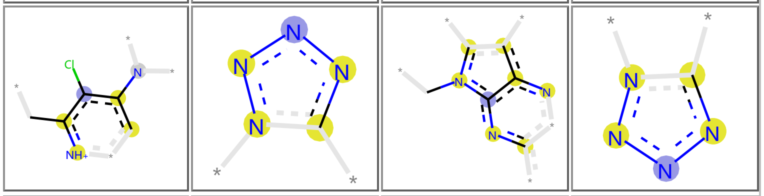
34	bit: 1959	zinc_freq: 4.5%	log2(FC): -1.85	mean_freq: 3.8%	active_freq: 1.7%	decoy_freq: 6.0%	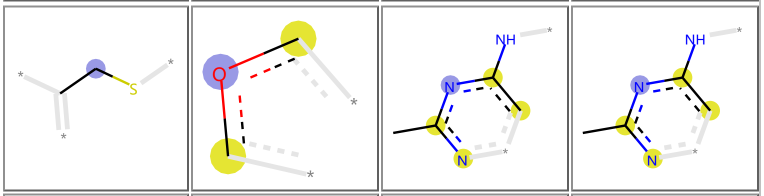
35	bit: 1853	zinc_freq: 4.4%	log2(FC): -1.47	mean_freq: 5.2%	active_freq: 2.8%	decoy_freq: 7.6%	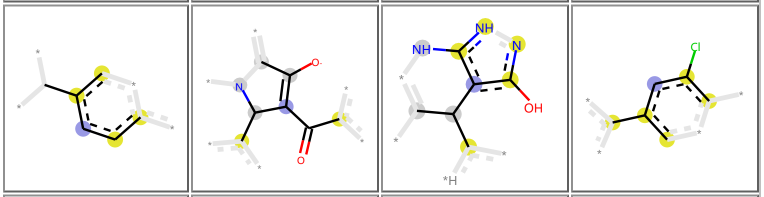
36	bit: 980	zinc_freq: 4.4%	log2(FC): 1.20	mean_freq: 5.0%	active_freq: 7.0%	decoy_freq: 3.0%	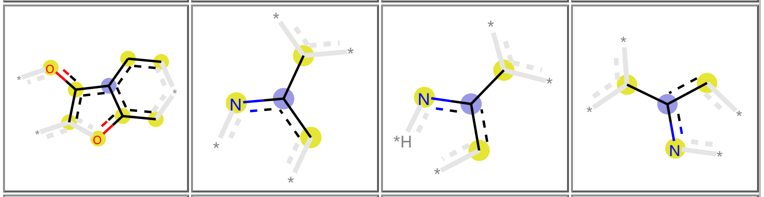
37	bit: 1791	zinc_freq: 4.4%	log2(FC): 1.05	mean_freq: 3.8%	active_freq: 5.1%	decoy_freq: 2.5%	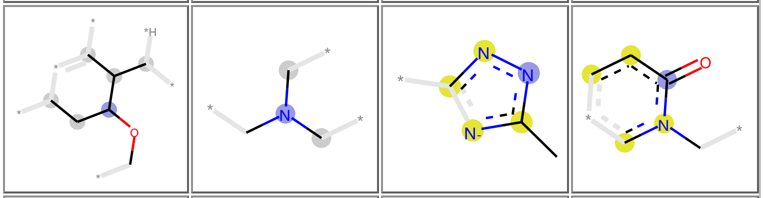
38	bit: 281	zinc_freq: 4.3%	log2(FC): -1.18	mean_freq: 3.9%	active_freq: 2.4%	decoy_freq: 5.5%	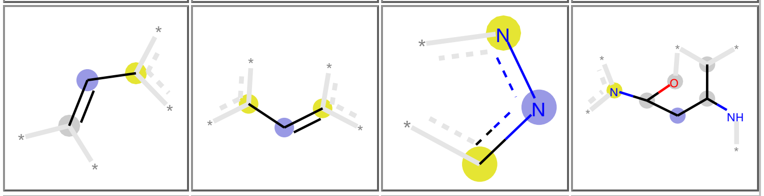
39	bit: 1405	zinc_freq: 4.1%	log2(FC): 1.02	mean_freq: 4.0%	active_freq: 5.4%	decoy_freq: 2.7%	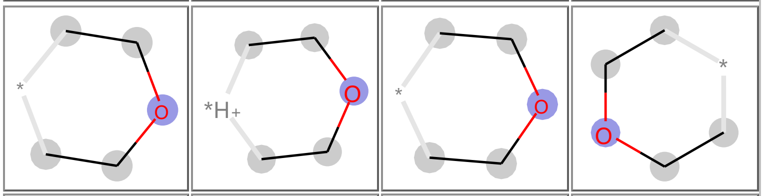
40	bit: 45	zinc_freq: 4.1%	log2(FC): 1.35	mean_freq: 4.9%	active_freq: 7.1%	decoy_freq: 2.8%	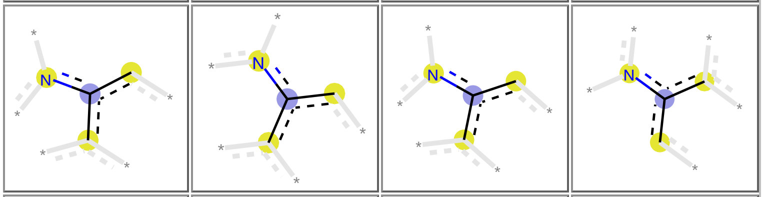
41	bit: 2031	zinc_freq: 4.1%	log2(FC): -1.35	mean_freq: 4.9%	active_freq: 2.7%	decoy_freq: 7.0%	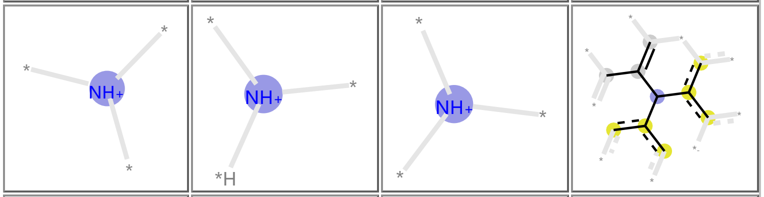
42	bit: 523	zinc_freq: 4.0%	log2(FC): 1.02	mean_freq: 3.9%	active_freq: 5.2%	decoy_freq: 2.6%	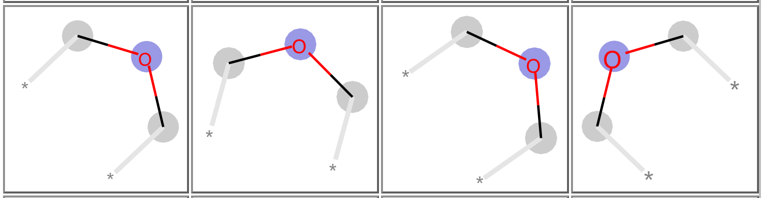
43	bit: 193	zinc_freq: 3.9%	log2(FC): 1.35	mean_freq: 4.3%	active_freq: 6.2%	decoy_freq: 2.4%	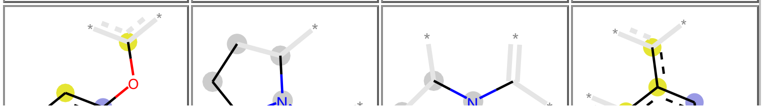

	bit	zinc_freq	log2_FC	mean_freq	active_freq	decoy_freq	
							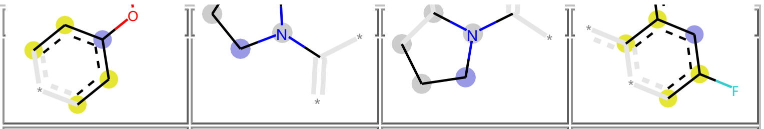
44	bit: 879	zinc_freq: 3.9%	log2(FC): -1.12	mean_freq: 4.6%	active_freq: 2.9%	decoy_freq: 6.2%	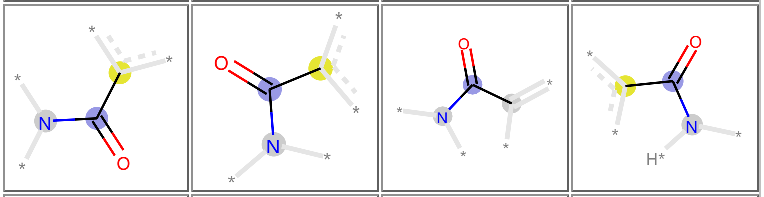
45	bit: 241	zinc_freq: 3.7%	log2(FC): -1.06	mean_freq: 3.3%	active_freq: 2.1%	decoy_freq: 4.5%	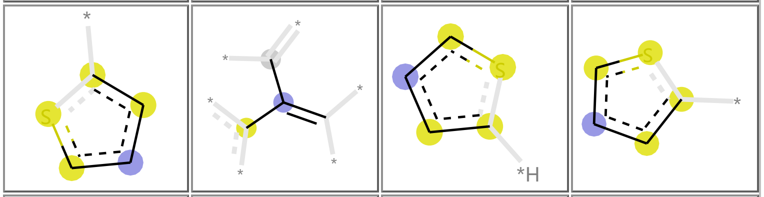
46	bit: 29	zinc_freq: 3.7%	log2(FC): 1.03	mean_freq: 3.9%	active_freq: 5.3%	decoy_freq: 2.6%	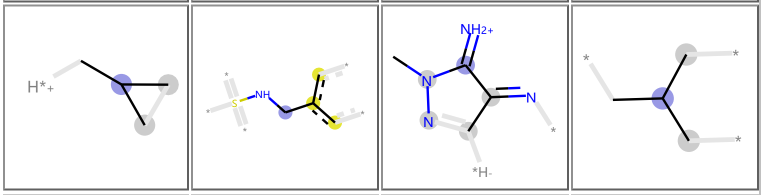
47	bit: 502	zinc_freq: 3.7%	log2(FC): -1.01	mean_freq: 3.6%	active_freq: 2.4%	decoy_freq: 4.8%	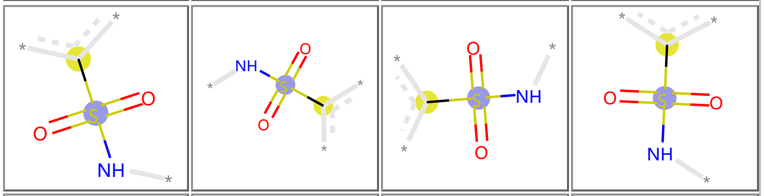
48	bit: 1043	zinc_freq: 3.6%	log2(FC): 1.31	mean_freq: 3.2%	active_freq: 4.6%	decoy_freq: 1.9%	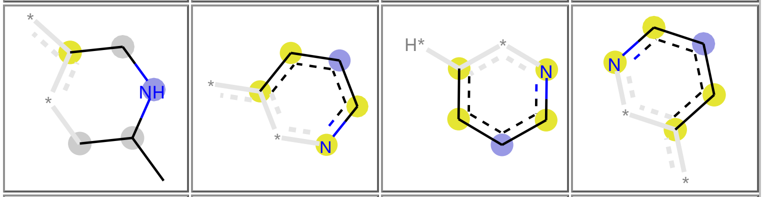
49	bit: 448	zinc_freq: 3.6%	log2(FC): 1.30	mean_freq: 3.3%	active_freq: 4.7%	decoy_freq: 1.9%	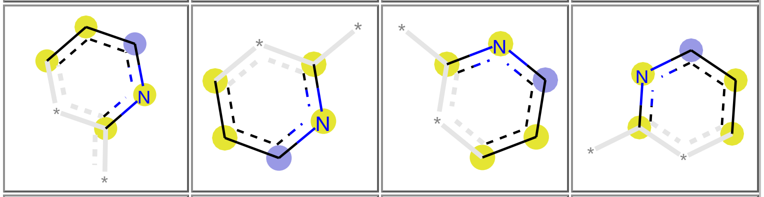
50	bit: 1034	zinc_freq: 3.3%	log2(FC): 1.37	mean_freq: 5.5%	active_freq: 7.9%	decoy_freq: 3.1%	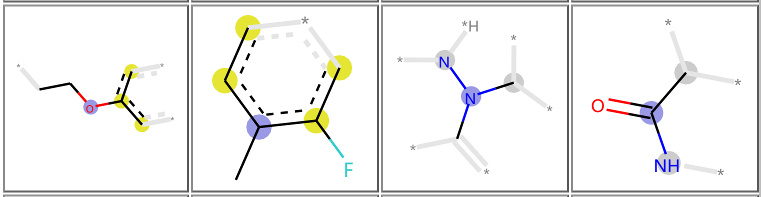
51	bit: 909	zinc_freq: 3.3%	log2(FC): -2.05	mean_freq: 4.1%	active_freq: 1.6%	decoy_freq: 6.6%	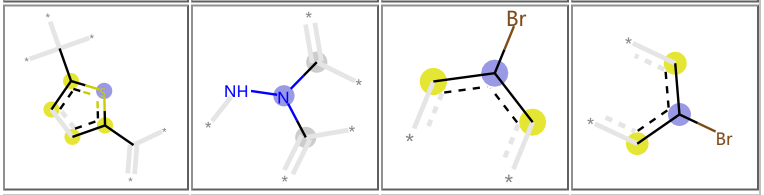
52	bit: 97	zinc_freq: 3.2%	log2(FC): -1.58	mean_freq: 3.4%	active_freq: 1.7%	decoy_freq: 5.1%	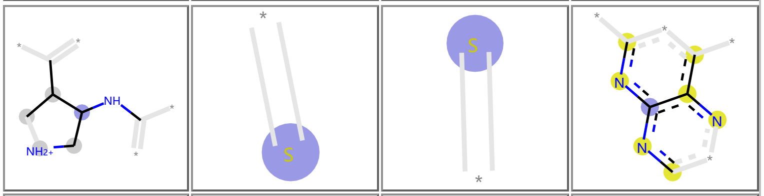
53	bit: 984	zinc_freq: 3.2%	log2(FC): 1.05	mean_freq: 4.7%	active_freq: 6.4%	decoy_freq: 3.1%	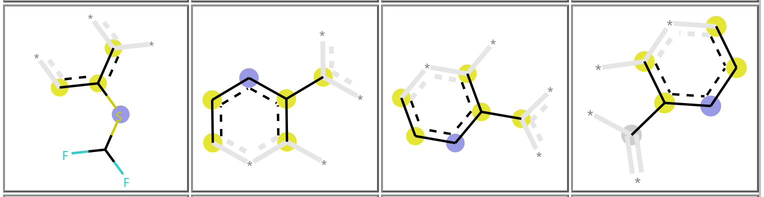
54							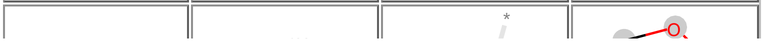

	bit	zinc_freq	log2_FC	mean_freq	active_freq	decoy_freq	
	bit: 1441	zinc_freq: 3.1%	log2(FC): 1.03	mean_freq: 3.3%	active_freq: 4.5%	decoy_freq: 2.2%	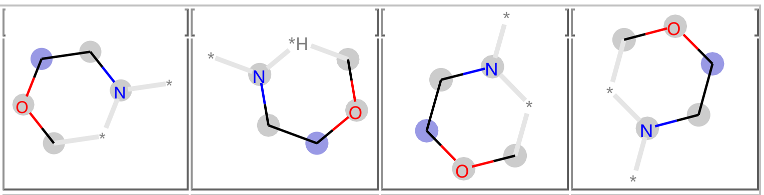
55	bit: 1157	zinc_freq: 3.0%	log2(FC): -1.01	mean_freq: 3.8%	active_freq: 2.5%	decoy_freq: 5.0%	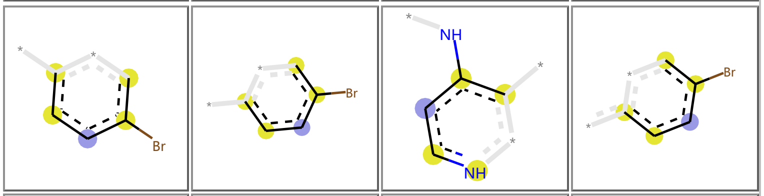
56	bit: 785	zinc_freq: 3.0%	log2(FC): 1.10	mean_freq: 6.0%	active_freq: 8.2%	decoy_freq: 3.8%	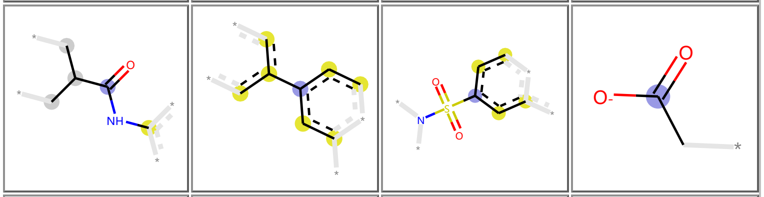
57	bit: 806	zinc_freq: 2.8%	log2(FC): 1.05	mean_freq: 5.0%	active_freq: 6.8%	decoy_freq: 3.3%	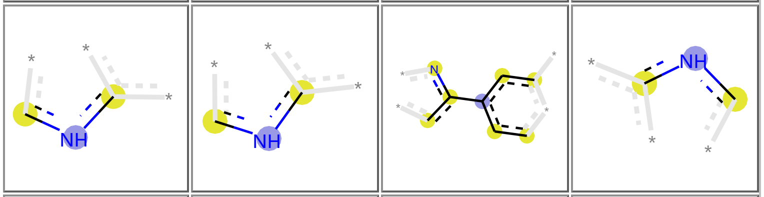
58	bit: 1427	zinc_freq: 2.8%	log2(FC): -1.91	mean_freq: 3.6%	active_freq: 1.5%	decoy_freq: 5.6%	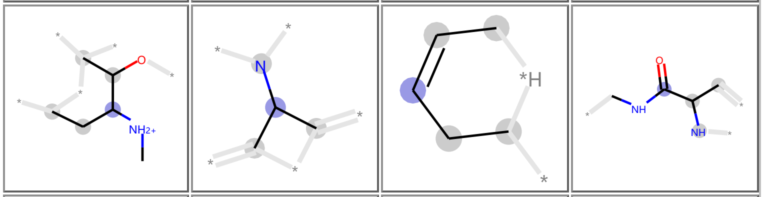
59	bit: 1416	zinc_freq: 2.8%	log2(FC): 1.05	mean_freq: 4.3%	active_freq: 5.9%	decoy_freq: 2.8%	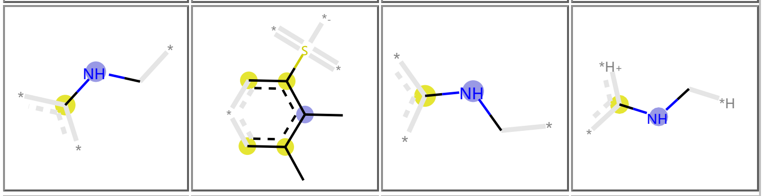
60	bit: 680	zinc_freq: 2.8%	log2(FC): 1.45	mean_freq: 4.7%	active_freq: 6.8%	decoy_freq: 2.5%	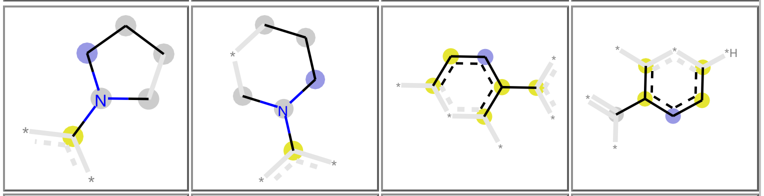
61	bit: 42	zinc_freq: 2.8%	log2(FC): -2.37	mean_freq: 3.2%	active_freq: 1.0%	decoy_freq: 5.3%	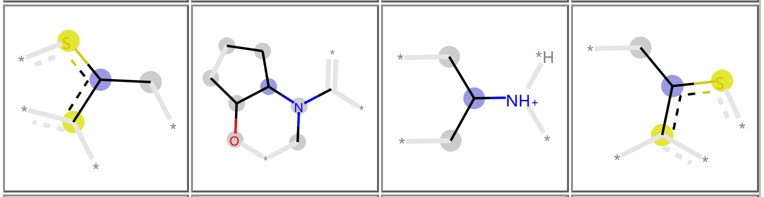
62	bit: 1501	zinc_freq: 2.6%	log2(FC): 1.02	mean_freq: 3.3%	active_freq: 4.5%	decoy_freq: 2.2%	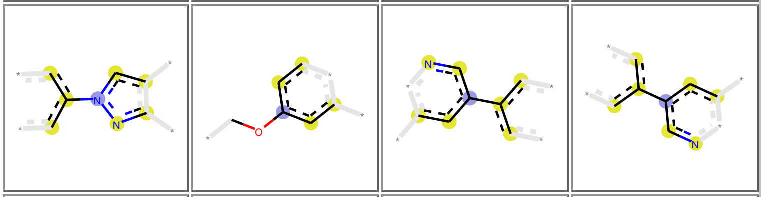
63	bit: 1586	zinc_freq: 2.6%	log2(FC): 2.19	mean_freq: 3.6%	active_freq: 6.0%	decoy_freq: 1.3%	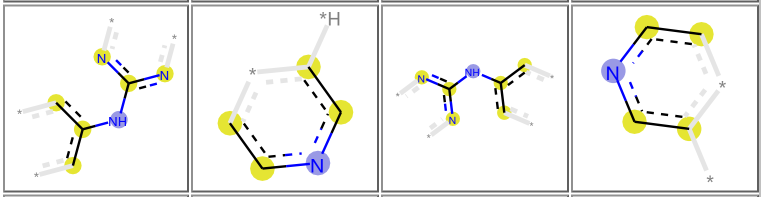
64	bit: 1507	zinc_freq: 2.4%	log2(FC): -1.58	mean_freq: 3.2%	active_freq: 1.6%	decoy_freq: 4.9%	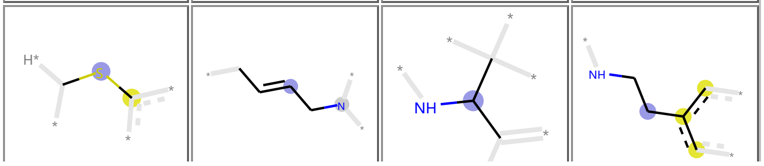

	bit	zinc_freq	log2_FC	mean_freq	active_freq	decoy_freq	
							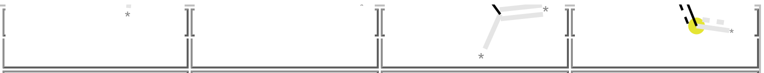
**65**	bit: 1045	zinc_freq: 2.4%	log2(FC): 1.52	mean_freq: 3.4%	active_freq: 5.1%	decoy_freq: 1.8%	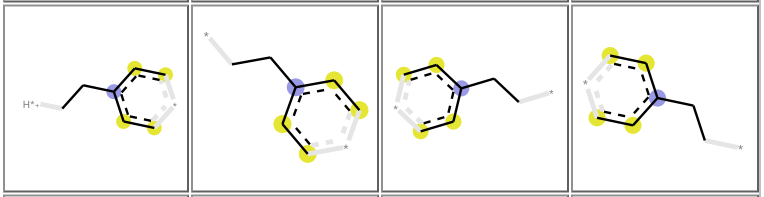
**66**	bit: 927	zinc_freq: 2.3%	log2(FC): -1.18	mean_freq: 3.0%	active_freq: 1.9%	decoy_freq: 4.2%	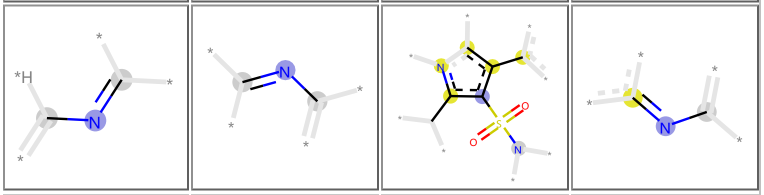
**67**	bit: 489	zinc_freq: 2.3%	log2(FC): 1.41	mean_freq: 4.3%	active_freq: 6.3%	decoy_freq: 2.4%	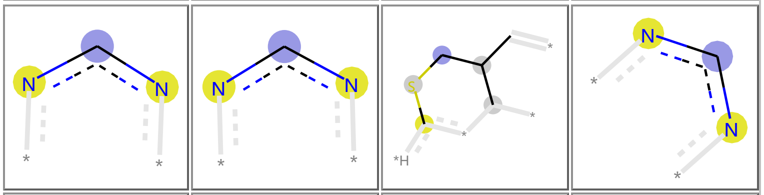
**68**	bit: 79	zinc_freq: 2.2%	log2(FC): 1.15	mean_freq: 3.9%	active_freq: 5.4%	decoy_freq: 2.4%	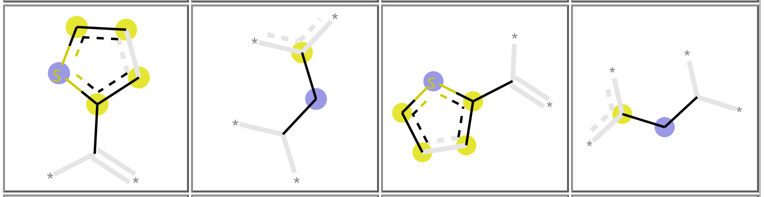
**69**	bit: 323	zinc_freq: 2.1%	log2(FC): 1.44	mean_freq: 4.6%	active_freq: 6.7%	decoy_freq: 2.5%	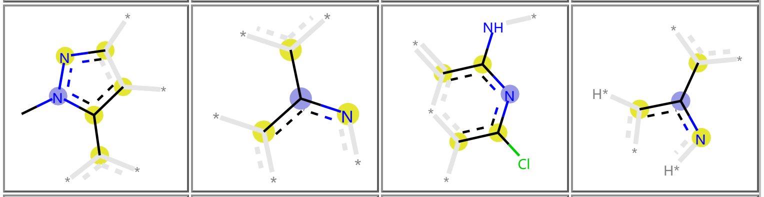
**70**	bit: 878	zinc_freq: 1.9%	log2(FC): 1.46	mean_freq: 4.3%	active_freq: 6.4%	decoy_freq: 2.3%	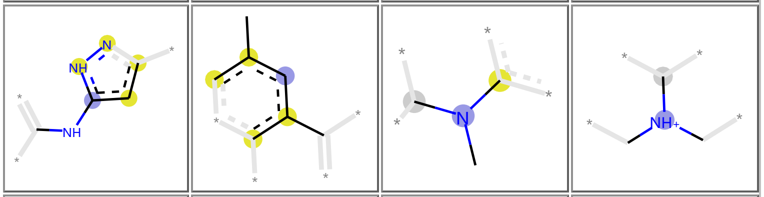
**71**	bit: 708	zinc_freq: 1.9%	log2(FC): 1.59	mean_freq: 4.2%	active_freq: 6.3%	decoy_freq: 2.1%	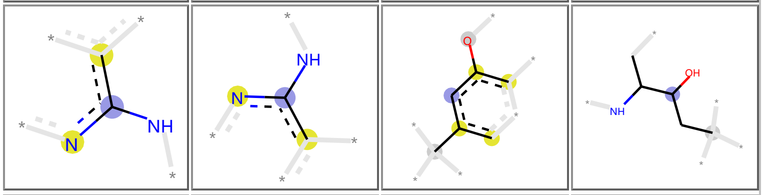
**72**	bit: 271	zinc_freq: 1.9%	log2(FC): -1.28	mean_freq: 3.7%	active_freq: 2.2%	decoy_freq: 5.3%	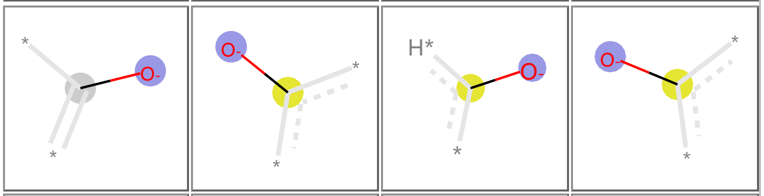
**73**	bit: 491	zinc_freq: 1.8%	log2(FC): 2.27	mean_freq: 5.7%	active_freq: 9.4%	decoy_freq: 1.9%	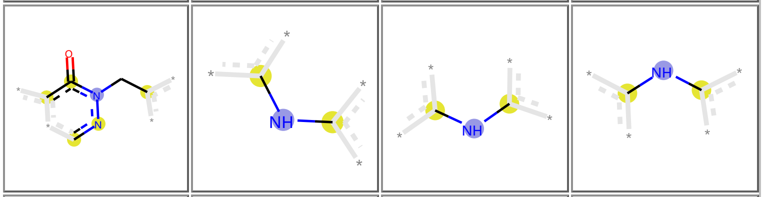
**74**	bit: 381	zinc_freq: 1.7%	log2(FC): 1.47	mean_freq: 4.6%	active_freq: 6.7%	decoy_freq: 2.4%	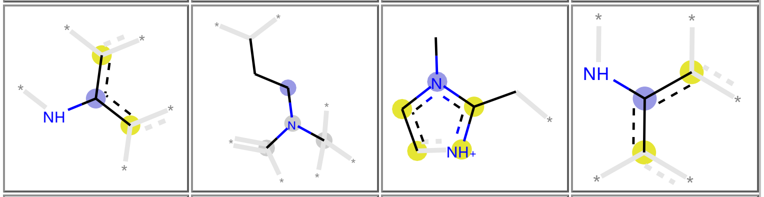
**75**	bit: 203	zinc_freq: 1.7%	log2(FC): 1.08	mean_freq: 3.0%	active_freq: 4.1%	decoy_freq: 2.0%	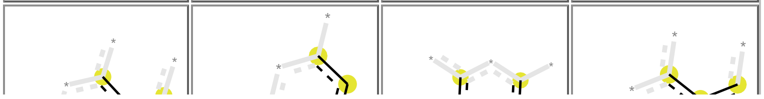

	bit	zinc_freq	log2_FC	mean_freq	active_freq	decoy_freq				
							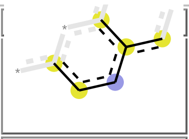	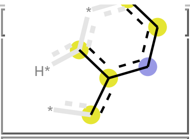	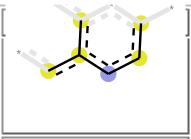	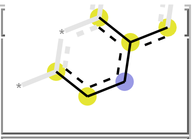
76	bit: 2010	zinc_freq: 1.7%	log2(FC): 1.57	mean_freq: 3.4%	active_freq: 5.1%	decoy_freq: 1.7%	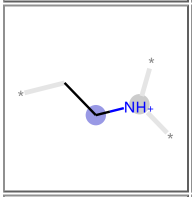	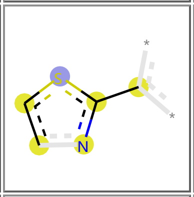	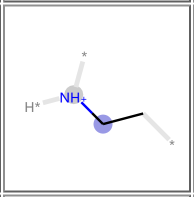	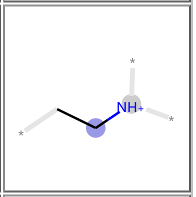
77	bit: 832	zinc_freq: 1.6%	log2(FC): 1.43	mean_freq: 3.1%	active_freq: 4.5%	decoy_freq: 1.7%	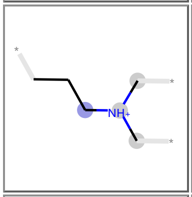	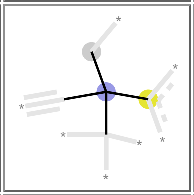	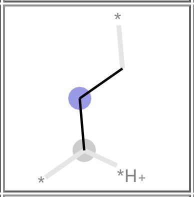	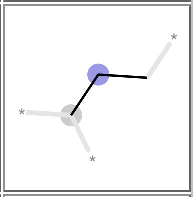
78	bit: 1747	zinc_freq: 1.5%	log2(FC): 1.30	mean_freq: 4.5%	active_freq: 6.5%	decoy_freq: 2.6%	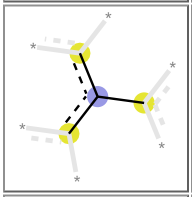	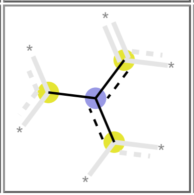	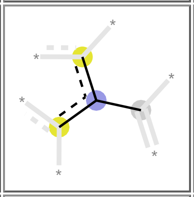	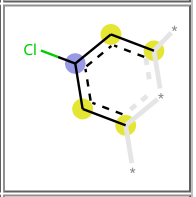
79	bit: 1733	zinc_freq: 1.5%	log2(FC): 1.12	mean_freq: 3.0%	active_freq: 4.1%	decoy_freq: 1.9%	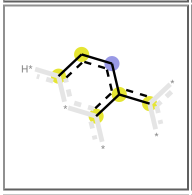	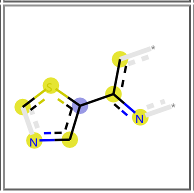	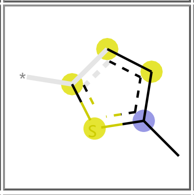	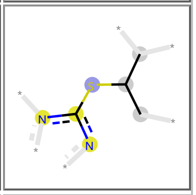
80	bit: 1313	zinc_freq: 1.4%	log2(FC): 1.51	mean_freq: 4.0%	active_freq: 5.9%	decoy_freq: 2.1%	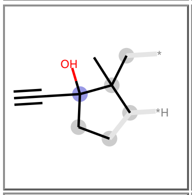	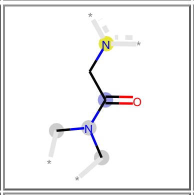	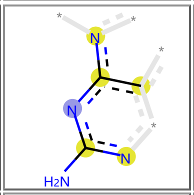	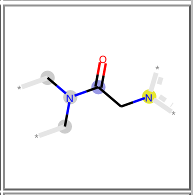
81	bit: 1617	zinc_freq: 1.4%	log2(FC): 1.84	mean_freq: 3.5%	active_freq: 5.5%	decoy_freq: 1.5%	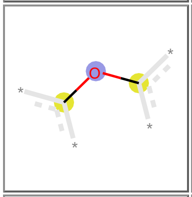	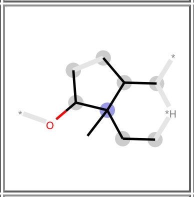	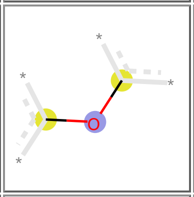	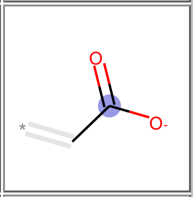
82	bit: 457	zinc_freq: 1.0%	log2(FC): 1.74	mean_freq: 6.2%	active_freq: 9.5%	decoy_freq: 2.9%	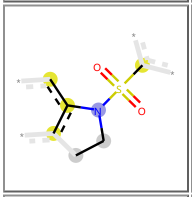	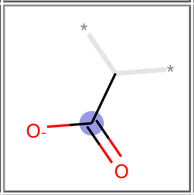	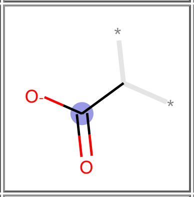	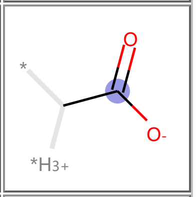
83	bit: 1459	zinc_freq: 0.9%	log2(FC): 1.32	mean_freq: 3.4%	active_freq: 4.8%	decoy_freq: 1.9%	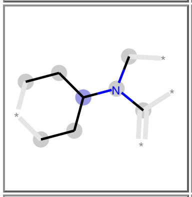	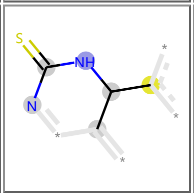	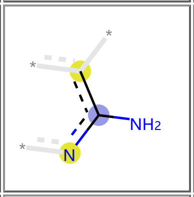	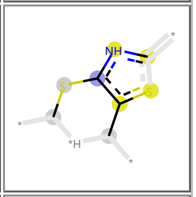
84	bit: 1868	zinc_freq: 0.8%	log2(FC): 1.94	mean_freq: 3.5%	active_freq: 5.6%	decoy_freq: 1.5%	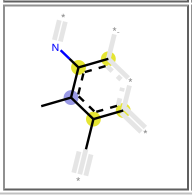	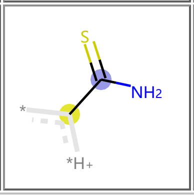	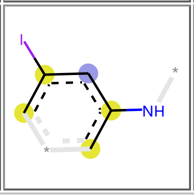	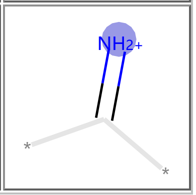
