# Supplementary material for: Predicting or Pretending: Artificial Intelligence for Protein-Ligand Interactions Lack of Sufficiently Large and Unbiased Datasets
Source: Front Pharmacol. 2020 Feb 25;11:69. doi: 10.3389/fphar.2020.00069 (PMC7052818; doi:10.3389/fphar.2020.00069)
Supplement: Supplementary file 2 [file DataSheet_2.pdf]

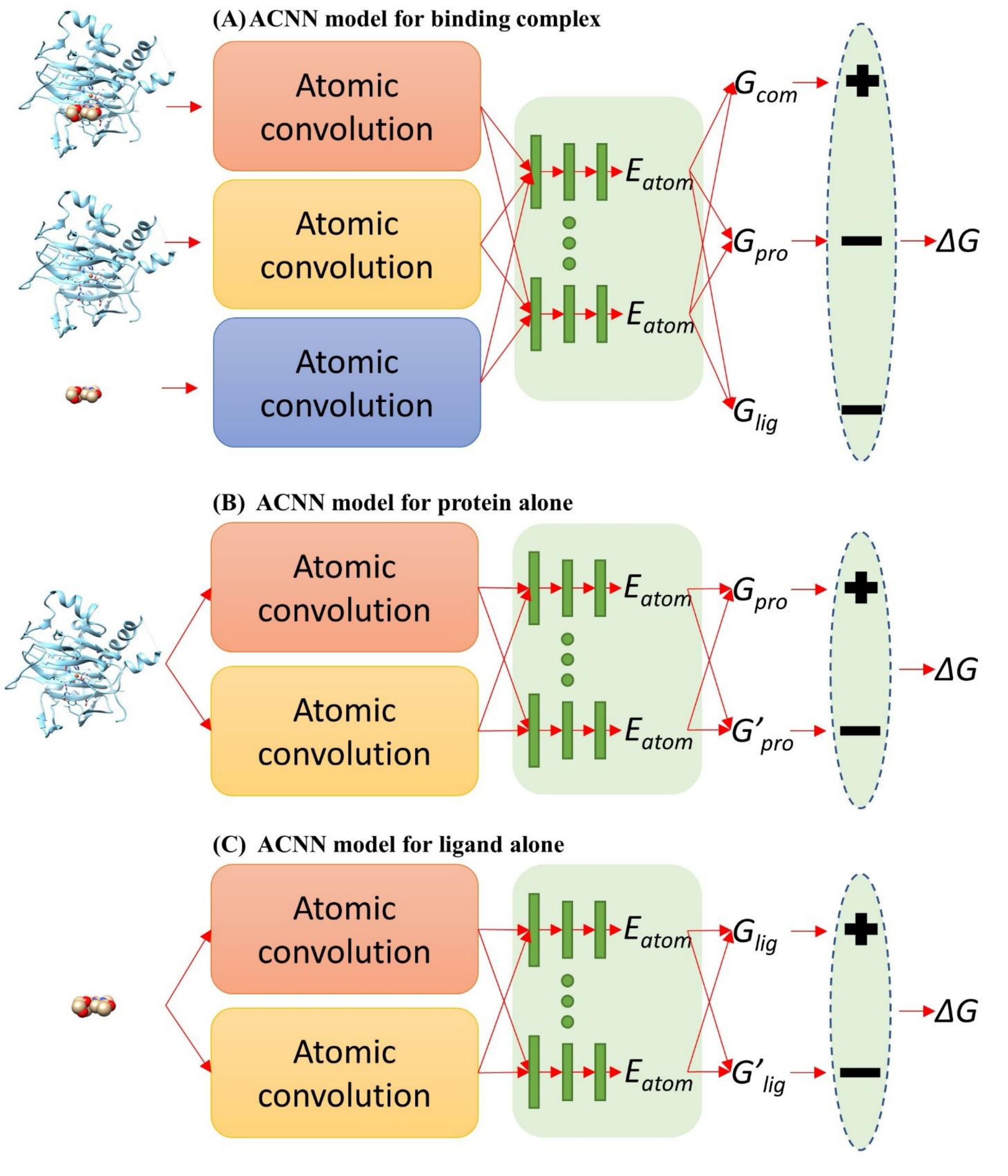

Supplementary Figure 1. Diagram of atomic convolutions neural network (ACNN) model for treating binding complex, protein alone and ligand alone, respectively. Each model has three (for binding complex) or two (for protein alone and ligand alone) independent atomic convolution blocks (only sharing initial parameters) and one weight-sharing atomistic fully connected layer.

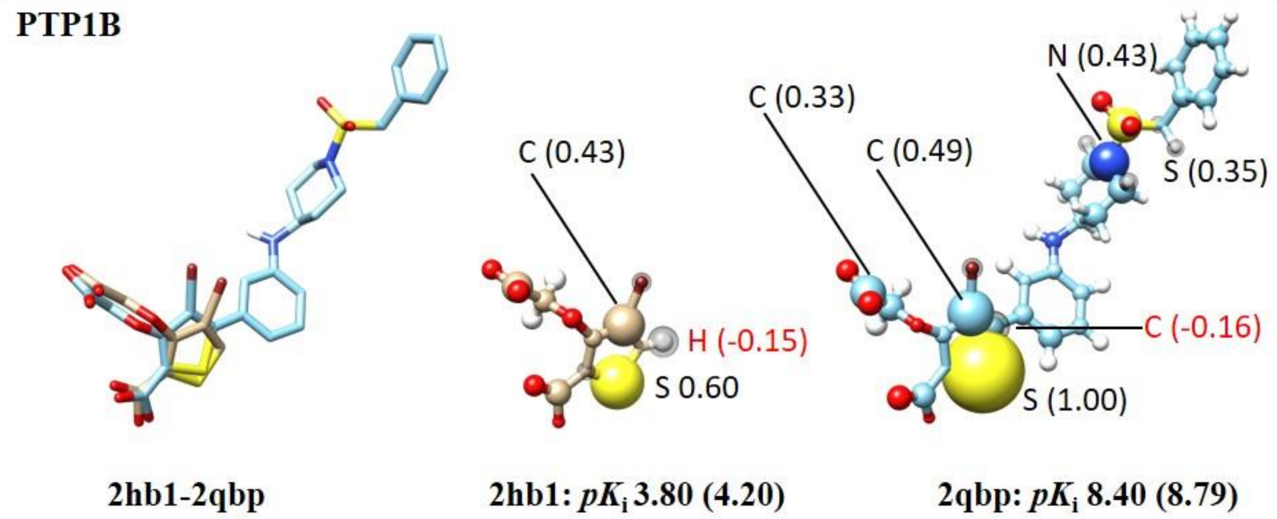

Supplementary Figure 2. Atomic contributions of PTP1B inhibitors derived from ACNN model (ligand alone) using a different random seed. The atomic contributions changed dramatically comparing with the values shown in Figure 2A. The first column shows the superimposed ligand structures using the binding pocket alignment approach. The second and third columns show atomic contributions of each ligand. The size of the balls represents the absolute values of atomic contributions. The atomic contributions of selected atoms are labeled explicitly. The atoms with black spheres have negative contributions. The molecular images were generated using UCSF Chimera (Pettersen et al., 2004).

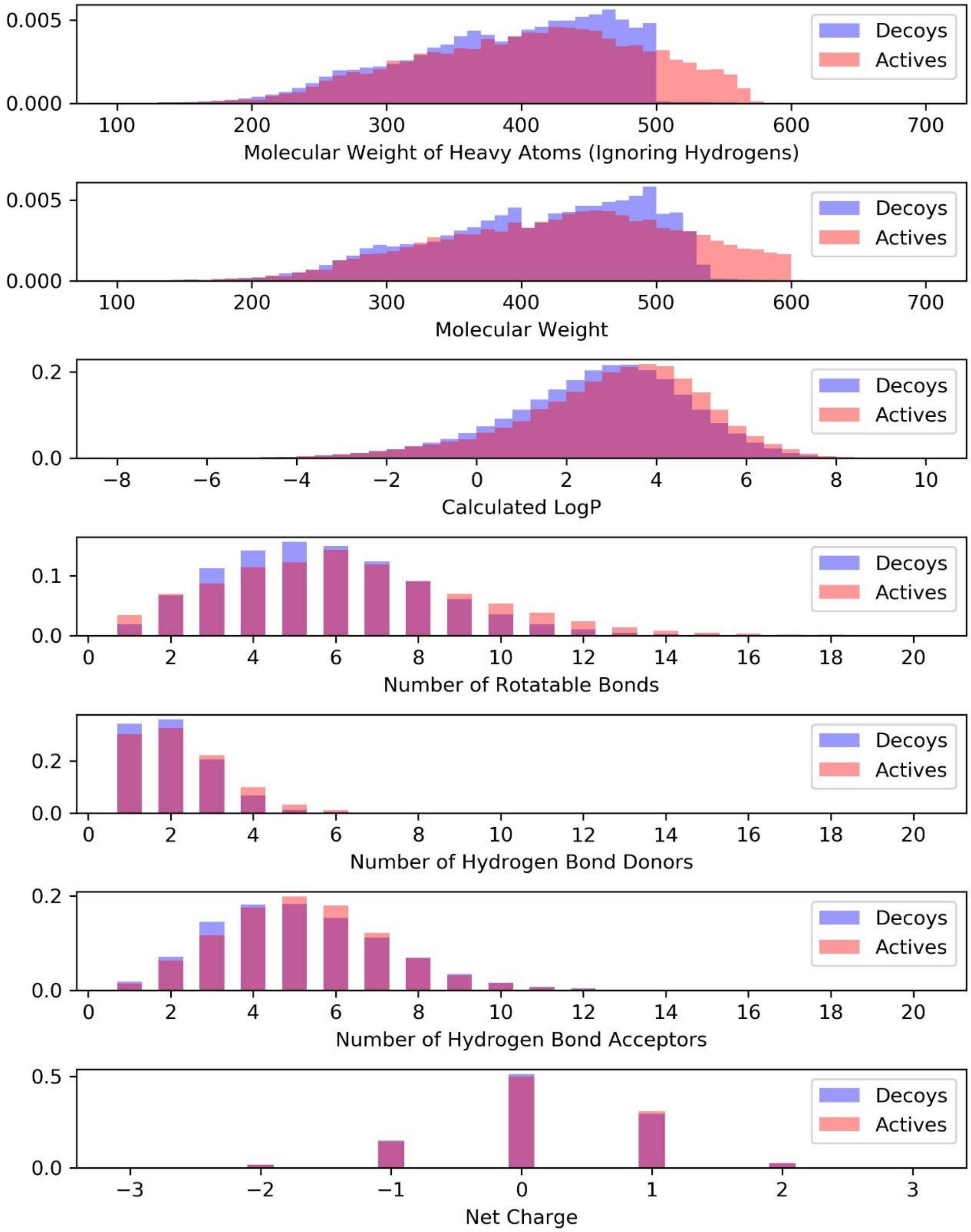

Supplementary Figure 3. Property distributions of all actives and decoys in DUD-E dataset. Actives are colored in red and decoys in blue.

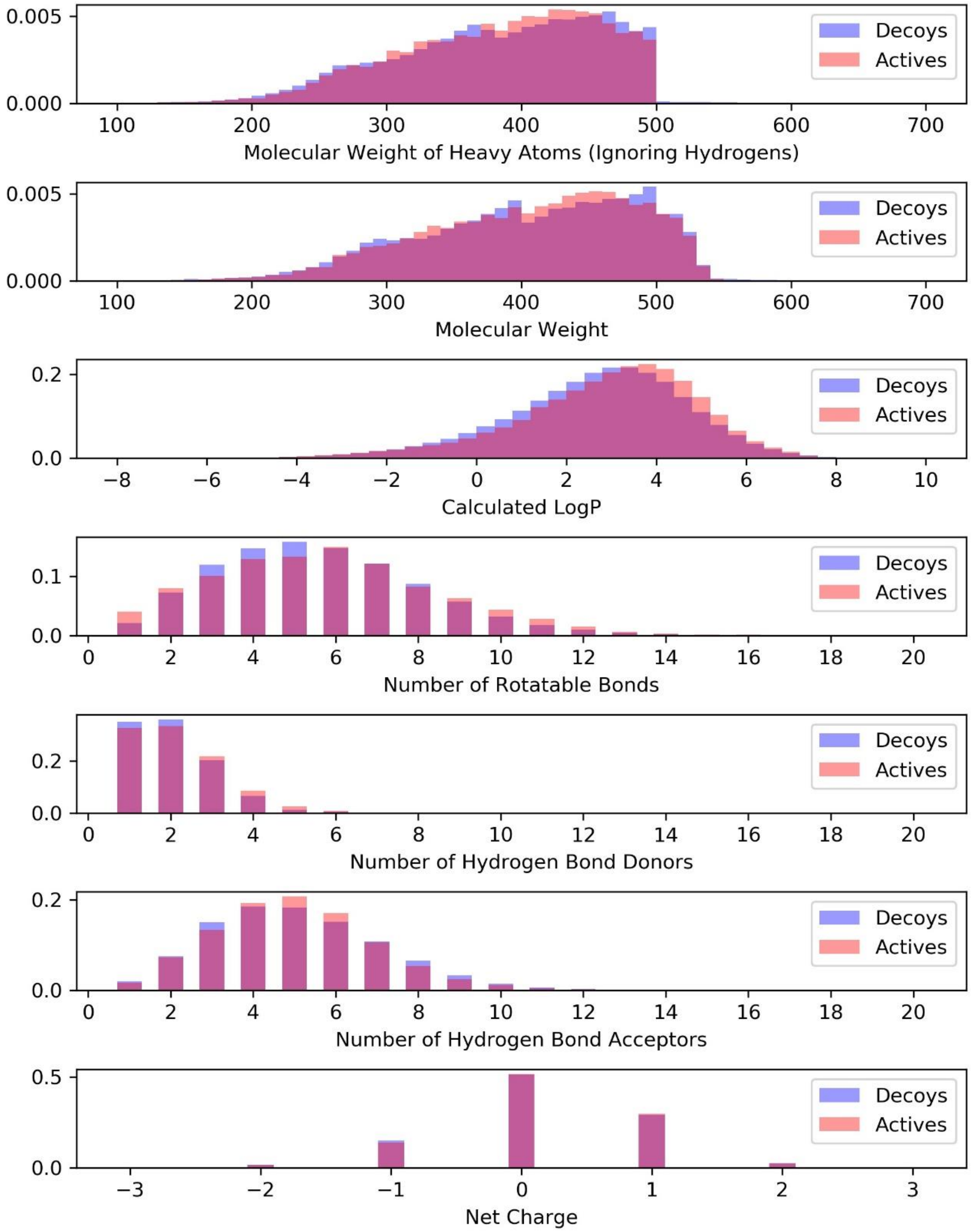

Supplementary Figure 4. Property distributions of all actives and decoys in the DUD-E$_{(MW\leq500)}$ dataset. Actives are colored in red and decoys in blue.

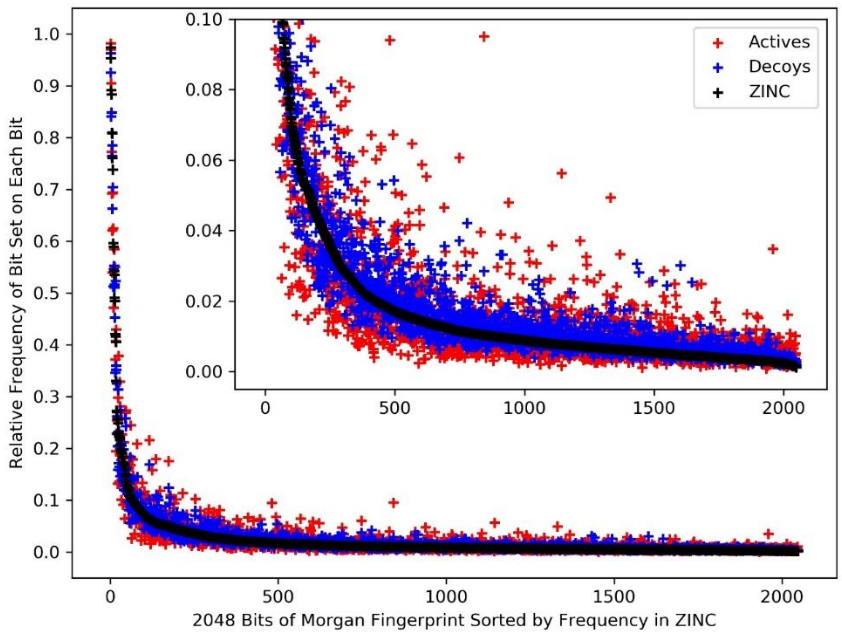

Supplementary Figure 5. Statistics of fingerprint bits on the DUD-$E_{(MW \leq 500)}$ dataset. Actives are colored in red, decoys in blue and ZINC compounds in black.

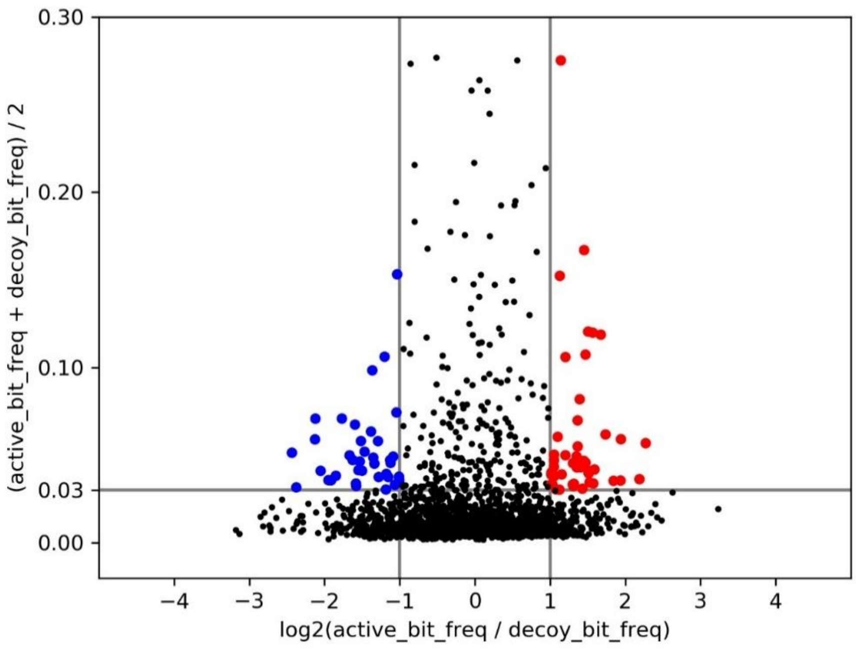

Supplementary Figure 6. Statistics of fingerprint bits on the DUD-E$_{(MW\leqslant500)}$ dataset. The red dots are the bits enrich in the actives, and the blue dots are bits enrich in the decoys.
